# Processing of DNA single-strand breaks with oxidatively damaged ends by LIG1

**DOI:** 10.1093/nar/gkaf1344

**Published:** 2025-12-10

**Authors:** Kanal E Balu, Danah Almohdar, Camden Lerner, Jacob Ratcliffe, Qun Tang, Tanay Parwal, Kar M Lee, Aishwarya Prakash, Melike Çağlayan

**Affiliations:** Department of Biochemistry and Molecular Biology, University of Florida, Gainesville, FL 32610, United States; Department of Biochemistry and Molecular Biology, University of Florida, Gainesville, FL 32610, United States; Department of Biochemistry and Molecular Biology, University of Florida, Gainesville, FL 32610, United States; Department of Biochemistry and Molecular Biology, University of Florida, Gainesville, FL 32610, United States; Department of Biochemistry and Molecular Biology, University of Florida, Gainesville, FL 32610, United States; Department of Biochemistry and Molecular Biology, University of Florida, Gainesville, FL 32610, United States; Department of Biochemistry and Molecular Biology, University of Florida, Gainesville, FL 32610, United States; University of South Alabama, Mitchell Cancer Institute, Department of Biochemistry and Molecular Biology, Mobile, AL 36604, United States; Department of Biochemistry and Molecular Biology, University of Florida, Gainesville, FL 32610, United States

## Abstract

DNA ligase 1 (LIG1) seals broken strand breaks by joining two adjacent ends during DNA replication and repair transactions. We previously reported atomic-level insight into the strategies that LIG1 uses to discriminate mismatches or ribonucleotides. However, how LIG1 processes strand breaks with oxidatively damaged ends in the absence and presence of a “wrong” sugar remains unknown. Here, we determined the crystal structures of LIG1/nick DNA complexes with 3′-8-oxodG and 3′-8-oxorG templating A or C during the pre- and post-catalytic steps of the ligation reaction. Our structures demonstrated differences in the distances at the +1 and +2 nucleotides relative to the 3′-end of the nick and a shift in the template base position to accommodate the oxidative lesion depending on the dual coding potential of 8-oxoG, which forms Hoogsteen or Watson–Crick base pairing in -*syn* or -*anti* conformation. Furthermore, these structural adjustments lead to mutagenic ligation or non-mutagenic end joining of the nick substrates. Overall, our findings provide mechanistic insight into how LIG1 processes nicks harboring oxidative damage and ribonucleotides to ensure fidelity at the final ligation step of DNA repair and replication to maintain genome integrity.

## Graphical Abstract

**Graphical Abstract:**
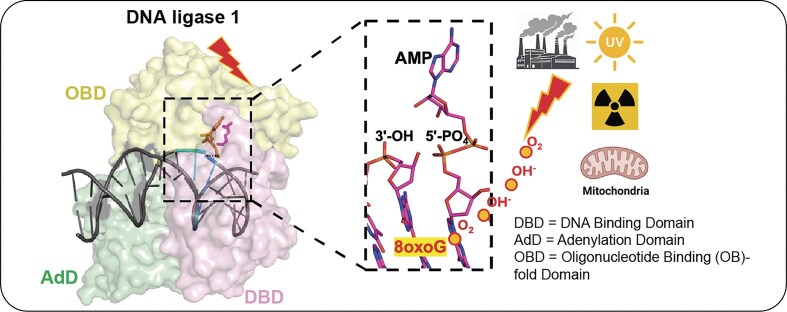

## Introduction

Genomic DNA can be oxidized by reactive oxygen species (ROS), which are generated as byproducts of oxygen respiration and due to various endogenous stressors or exposure to environmental hazards such as ionizing radiation and chemical toxicants [1–3]. Guanine is particularly susceptible to oxidation, and 8-oxo-2′-deoxyguanine (8-oxoG) is the most abundant and best characterized oxidized guanine base [4, 5]. The coding potential of 8-oxoG is dictated by two distinct conformations of the oxidized base that can pair with cytosine (C) in -*anti* conformation through Watson–Crick base pairing and adopt -*syn* conformation when paired with adenine (A) through Hoogsteen base pairing [6]. DNA precursor nucleotides (dNTPs) in the cellular nucleotide pool are subject to oxidative damage and are more vulnerable to endogenous and exogenous oxidants than DNA protected by chromatin [7, 8]. Similarly, dGTP in the nucleotide pool is most frequently oxidized to form 7,8-dihydro-8′-oxo-dGTP (8-oxodGTP), which can be incorporated into the genome opposite either A (mutagenic) or C (non-mutagenic), where the former is implicated in driving tumorigenesis, causing A–T to C–G transversions [9–11]. Furthermore, ribonucleotides (rNTPs), the most abundant noncanonical nucleotides, can be frequently incorporated into the genome by DNA polymerases at a remarkably high rate during DNA replication and repair, impacting genome stability [12]. The oxidation of the rNTP pool by ROS generates oxidized ribonucleotides, and rGTP is analogously oxidized to 7,8-dihydro-8-oxo-guanosine (8-oxorGTP), which can constitute up to 5% of the rGTP pool [13–16]. Moreover, even relatively low oxidative stress conditions in human cells result in the oxidation of 0.2%–5% of free rGTP into 8-oxorGTP [17–20]. To sanitize the oxidized nucleotide pool, MutT homolog 1 (MTH1) hydrolyzes them into monophosphate forms, and tumors sustain higher MTH1 levels than adjacent normal tissues do, as observed through global gene expression profiling in cancer patient datasets [21, 22]. The depletion or chemical inhibition of MTH1 has been reported to increase 8-oxodG incorporation into the genome and induce genomic DNA breaks in cancer cells [23]. MTH1 exhibits substrate specificity for 8-oxodGTP, and the enzymes that process DNA damage, such as 8-oxoguanine glycosylase (OGG1), RNase H2, and ribonucleotide reductase, exhibit weak activity for 8-oxorGTP or 8-oxorG [24, 25].

Oxidized nucleotides can interfere with faithful DNA replication and transcription, leading to genome instability and the induction of various cellular abnormalities, such as mutations, cancer, neurological diseases, and cellular senescence [8–11]. During times of oxidative stress, DNA polymerases from the A, B, C, X, and Y families can perform futile DNA synthesis by inserting 8-oxodGTP and 8-oxorGTP, preferentially opposite a favorable template base A, with different catalytic efficiency and fidelity [26–36]. The incorporation of oxidized dNTPs or rNTPs by error-prone DNA polymerases could lead to the formation of repair intermediates containing 8-oxodG or 8-oxorG at the 3′-end of the nick, resulting in error-prone versus error-free repair outcomes at the final step when DNA ligases join broken strands to complete the process [37–39].

Manifesting essential roles in nuclear replication, DNA repair, and recombination, human DNA ligases, DNA ligase 1 (LIG1), DNA ligase 3α (LIG3α), and DNA ligase 4 (LIG4), catalyze a conserved ligation reaction involving three sequential chemical steps by utilizing the energy of ATP [40]: the formation of a ligase:adenylate intermediate after the active-site lysine residue attacks and forms a covalent bond with the α-phosphate of ATP in step 1, which is followed by the transfer of AMP from the adenylated ligase to the 5′-PO_4_ end of the nick, generating a 5′-adenylated DNA intermediate (DNA–AMP) in step 2. Finally, the 3′-OH terminus attacks the 5′-PO_4_ terminus downstream of the nick so that phosphodiester bond formation occurs between adjacent DNA ends, which is coupled to the release of AMP in step 3.

LIG1, the main replicative ligase, serves a critical role in the maturation of Okazaki fragments by ligating >50 million nicks during DNA replication and finalizes DNA excision repair pathways by an ultimate ligation step [41–43]. LIG1 employs a Mg^2+^-reinforced DNA binding mode to ensure fidelity, which is governed by two conserved glutamate residues, Glu(E)346Ala(A) and Glu(E)592Ala(A) [44]. We previously determined the X-ray structures of LIG1/nick DNA complexes with mismatches and revealed ligase strategies that favor or deter the ligation of base substitution errors incorporated by DNA polymerases [45]. Our structures demonstrated that the LIG1 active site can accommodate G:T mismatch in a similar conformation as canonical A:T, while it remains in the initial LIG1 adenylate form in the presence of A:C mismatch. We further demonstrated that LIG1 can efficiently seal nick DNA containing a single ribonucleotide at the 3′ end, and our structures of LIG1/3′-RNA–DNA hybrids provided an atomic insight into ribonucleotide selectivity through a network of interactions between critical active-side residues (Asp570 and Arg871) and the 2′-OH of the ribose at the nick [46, 47]. However, how LIG1 processes nick DNA harboring oxidative damage in the absence and presence of a single ribonucleotide at the 3′-end depending on the dual coding potential of 8-oxoG in a mutagenic -*syn* or error-free -*anti* conformation remains largely unknown.

In the present study, we determined the structures of LIG1/nick DNA complexes containing 8-oxodG and 8-oxorG at the 3′ strand, opposite to those of either templating A or C, during the pre- and post-catalytic steps of the ligation reaction, indicating the formation of a DNA–AMP intermediate and a phosphodiester bond formation, respectively. Our structures demonstrated a movement within the phosphodiester backbone at +2 nucleotides relative to the 3′-OH of the nick and revealed a shift in the template base depending on 8-oxodGTP(*anti*):C(*anti*) and 8-oxodGTP(*syn*):A(*anti*), which respectively form non-mutagenic Watson–Crick and mutagenic Hoogsteen base pairing. Moreover, we showed the mutagenic ligation of nick DNA substrates containing 3′-8-oxodG and 3′-8-oxorG templating A, whereas LIG1 exhibits substrate specificity for nicks containing 3′-8-oxorG over 3′-8-oxodG templating C. Finally, our results demonstrated an interplay between LIG1 and AP-Endonuclease 1 (APE1) during nick sealing coupled with proofreading of DNA repair intermediates containing ribonucleotides and oxidative damage at the 3′-end. Overall, our findings contribute to understanding of the mechanism by which LIG1 maintains fidelity depending on the dual coding potential of the oxidative lesion at nick and how LIG1 and APE1 coordinate to process mutagenic DNA intermediates at the final steps of the repair process.

## Methods

### Protein purifications

DNA ligase 1 (LIG1) protein (pET-24b) was purified as reported [48]. Briefly, the protein with 6×-His tag was overexpressed in BL21(DE3) *Escherichia coli* cells in Terrific Broth (TB) media with kanamycin (50 μg ml^−1^) and chloramphenicol (34 μg ml^−1^) at 37°C. The cells were induced with 0.5 mM isopropyl β-D-thiogalactoside when an OD_600_ of 1.0 was reached during protein overexpression overnight at 28°C and were then collected in the lysis buffer containing 50 mM Tris–HCl (pH 7.0), 500 mM NaCl, 20 mM imidazole, 10% glycerol, 1 mM phenylmethylsulfonyl fluoride, and an ethylenediaminetetraacetic acid (EDTA)-free protease inhibitor cocktail tablet by sonication at 4°C. The cell lysate was clarified at 31 000 × *g* for 1 h at 4°C. LIG1 protein was purified by a HisTrap HP column with an increasing imidazole concentration (20–300 mM) in the binding buffer containing 50 mM Tris–HCl (pH 7.0), 500 mM NaCl, 20 mM imidazole, and 10% glycerol at 4°C. The collected fractions were subsequently loaded on a HiTrap Heparin column in the binding buffer containing 50 mM Tris–HCl (pH 7.0), 50 mM NaCl, 1 mM EDTA, 1 mM dithiothreitol (DTT), and 10% glycerol, and then eluted with a linear gradient of NaCl up to 1 M. LIG1 protein was further purified by a Superdex 200 10/300 column in the buffer containing 20 mM Tris–HCl (pH 7.0), 200 mM NaCl, and 1 mM DTT. LIG1 wild-type and the mutant carrying amino acid substitutions at Glu(E)346 and Glu(E)592 residues to alanine (A), leading to ablation of the high-fidelity site (referred as EE/AA) were purified similarly.

AP-endonuclease 1 (APE1) protein (pET-24b) was purified as reported [45]. Briefly, the protein with 6×-His tag was overexpressed in BL21(DE3) *E. coli* cells, and the cells were harvested and lysed at 4°C, and the supernatant was loaded onto a HisTrap HP column as described earlier. APE1 protein was purified with an increasing imidazole gradient (0–300 mM) elution, loaded onto a HiTrap Heparin column as a linear gradient of NaCl up to 1 M, and then finally loaded on a Superdex 200 Increase 10/300 column in the buffer containing 50 mM Tris–HCl (pH 7.0), 500 mM NaCl, 5% glycerol, and 1 mM DTT. All proteins were concentrated and stored at -80°C. Protein quality was evaluated by running the proteins on a 10% sodium dodecyl sulfate–polyacrylamide gel electrophoresis (SDS–PAGE) gel, and the protein concentration was determined using A280.

### Crystallization and structure determination

LIG1 C-terminal (△261) EE/AA (E346A and E592A) mutant was used for crystallization due to its tendency to yield reproducible diffraction-quality crystals as reported previously [45–47]. LIG1^EE/AA^ protein was purified similarly as described earlier until the elution step in the buffer containing 1.0 mM EDTA to remove the metal ions. For crystallization, we also used LIG1 wild-type (LIG1^WT^) protein after its pretreatment with EDTA during purification. In both cases, LIG1 protein at 27 mg ml^−1^ concentration was mixed with the nick DNA substrates (Supplementary Table S1). Briefly, LIG1/DNA complex solution was prepared in the solution containing 20 mM Tris–HCl (pH 7.0), 200 mM NaCl, 1 mM DTT, 1 mM EDTA, and 1 mM ATP at 1.4:1 DNA:protein molar ratio and then mixed with 1 μl of reservoir solution (Supplementary Table S2). LIG1-nick DNA complex crystals were grown at 20°C using the hanging drop method, harvested, and submerged in the cryoprotectant solution containing reservoir solution mixed with 20% glycerol before being flash-cooled in liquid nitrogen for data collection. The crystals were kept at 100 K during X-ray diffraction data collection using the beamlines CHESS-7B2 and APS 22-ID. X-ray diffraction data reduction and scaling were performed using HKL2000 (HKL Research, Inc). All structures were solved by molecular replacement using PHASER with PDB entry 7SUM as a search model. Iterative rounds of model building were performed in COOT, and the final models were refined with PHENIX [45–47]. All structural images were drawn using PyMOL (The PyMOL Molecular Graphics System, V0.99, Schrödinger, LLC).

### DNA ligation assays

DNA ligation assays were performed to evaluate substrate efficiency of LIG1 (wild-type and EE/AA mutant) as reported [48]. We used nick DNA substrates containing 3′-8-oxodG:A, 3′-8-oxodG:C, 3′-8-oxorG:A, and 3′-8-oxorG:C (Supplementary Table S3). In control assays, we compared ligation efficiency in the presence of nick DNA substrate containing a canonical 3′-dG:C. The ligation reaction containing 50 mM Tris–HCl (pH: 7.5), 100 mM KCl, 10 mM MgCl_2_, 1 mM ATP, 1 mM DTT, 100 µg ml^−1^ bovine serum albumin (BSA), 1% glycerol, and nick DNA substrate (500 nM) was initiated by the addition of LIG1 (100 nM). The reaction samples were incubated at 37°C, stopped by quenching with an equal amount of the buffer containing 95% formamide, 20 mM EDTA, 0.02% bromophenol blue, and 0.02% xylene cyanol, and collected at the time points indicated in the figure legends. The reaction products were then separated by electrophoresis on an 18% denaturing polyacrylamide gel. The gels were scanned with a Typhoon Phosphor Imager (Amersham Typhoon RGB), and the data were analyzed using ImageQuant software. DNA ligation assays in the absence of Mg^2+^ were performed similarly in the reaction buffer containing 50 mM Tris–HCl (pH: 7.5), 100 mM KCl, 1 mM ATP, 1 mM DTT, 100 µg ml^−1^ BSA, 1% glycerol, nick DNA substrate (500 nM), and LIG1 (100 nM) to test the impact of the metal ion on nick sealing efficiency.

### APE1 assays

Exonuclease assays were performed to substrate specificity of APE1 and to evaluate APE1 and LIG1 activities simultaneously in the same reaction mixtureas reported [48]. In both cases, we used nick DNA substrates containing 3′-8-oxodG:A, 3′-8-oxodG:C, 3′-8-oxorG:A, and 3′-8-oxorG:C (Supplementary Tables S4 and S5). For both assays, the reaction mixture contains 50 mM Tris–HCl (pH 7.5), 100 mM KCl, 10 mM MgCl_2_, 1 mM ATP, 1 mM DTT, 100 µg ml^−1^ BSA, 1% glycerol, and DNA substrate (500 nM) in the final volume of 10 µl. The reaction was initiated by the addition of APE1 alone or the preincubated APE1/LIG1 protein mixture (100 nM). The reaction mixtures were incubated at 37°C for the time points as indicated in the figure legends. The reaction products were then mixed with an equal amount of gel loading buffer, separated by electrophoresis on 18% urea-PAGE gel, and the data were analyzed as described earlier.

### Nick DNA binding measurements by BioLayer interferometry assay

DNA binding kinetics of LIG1 were measured by BioLayer interferometry assays in real time using the nick DNA substrate with 3′-biotin label (Supplementary Table S6). Streptavidin biosensors were used to attach the biotin-labeled DNA and were hydrated at 20°C for 20 min in the buffer containing 50 mM Tris–HCl (pH 7.5), 100 mM KCl, and 1 mM DTT. The sensors were then immersed in DNA (40 nM) in the kinetics buffer containing 20 mM HEPES (pH 7.4), 200 mM NaCl, 0.5% BSA, and 0.05% Tween 20 for 300 s. After recording an initial baseline, the sensors with DNA were exposed to an increasing amount of LIG1 (wild-type or EE/AA mutant) at the concentration range indicated in the figure legends. DNA binding was performed for 240 s for association, and then in the kinetics buffer for 240 s for dissociation. In all measurements, the affinity constants (*K*_D_), the association (*k*_on_) and dissociation (*k*_off_) rates were calculated using the ForteBio Data Analysis software with 1:1 binding model. All images were drawn using Graph Pad Prism 7.

## Results

### Structures of LIG1 probe the mechanism underlying the processing of nick with ribonucleotides and oxidatively damaged ends

**Figure 1.**
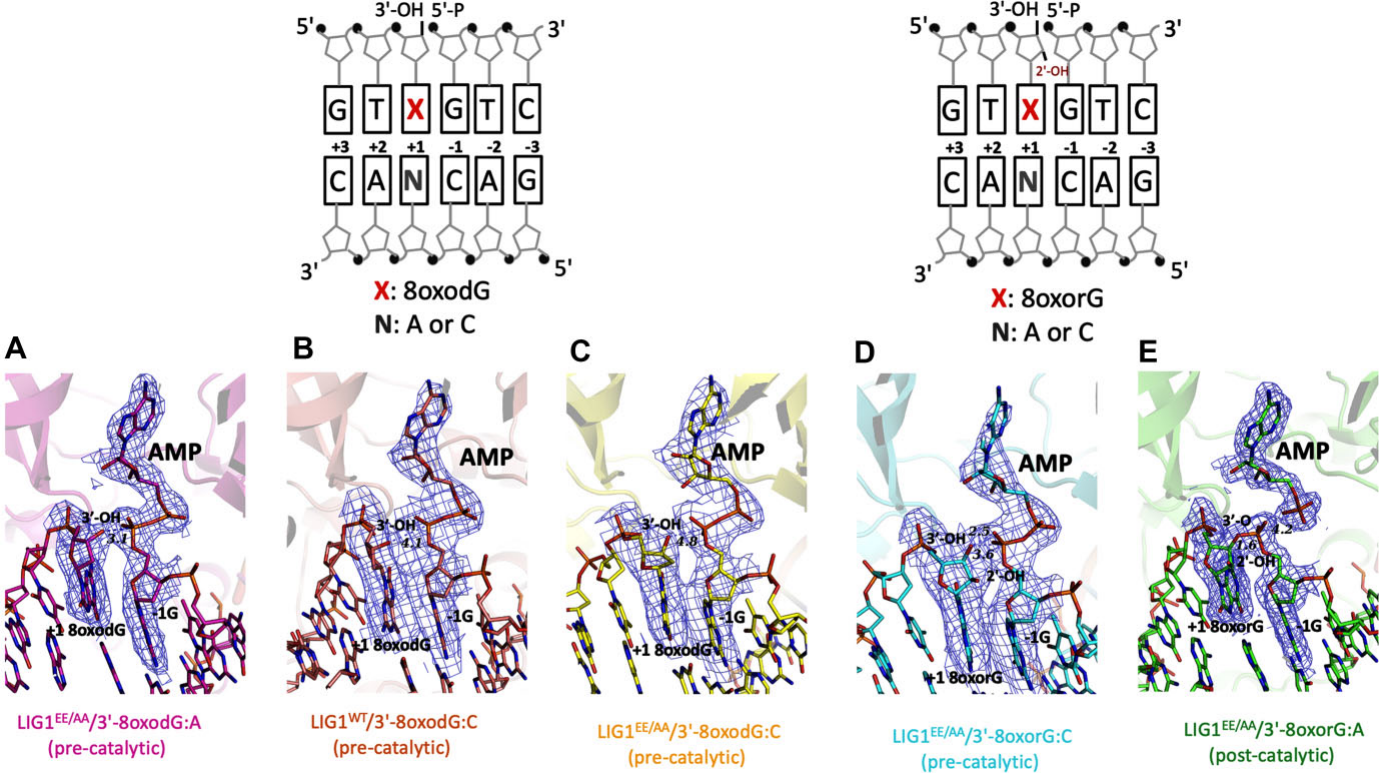
Structures of LIG1 bound to nick duplexes with oxidatively damaged ends. Structures for LIG1^EE/AA^/3′-8-oxodG:A (**A**), LIG1^WT^/3′-8-oxodG:C (**B**), LIG1^EE/AA^/3′-8-oxodG:C (**C**), and LIG1^EE/AA^/3′-8-oxorG:C (**D**) were captured at pre-catalytic step of the ligation reaction. LIG1^EE/AA^/3′-8-oxorG:A structure was captured at post-catalytic step of the ligation reaction (**E**). Schematic view of DNA substrate used in LIG1 crystallization shows the sequence of 3′- and 5′-ends at nick site.

We determined the structures of LIG1 in complex with nick DNA containing 3′-8-oxodG and 3′-8-oxorG opposite either A or C in the template position (Table 1). LIG1 wild-type (LIG1^WT^) and EE/AA mutant (LIG1^EE/AA^) were used for crystallization as reported previously [44–47]. We solved the LIG1/nick complexes containing 3′-8-oxodG:A (Fig. 1A), 3′-8-oxodG:C (Fig. 1B and C), and 3′-8-oxorG:C (Fig. 1D) at the pre-catalytic step of the ligation reaction referring to the formation of a DNA–AMP intermediate. However, we determined the LIG1/nick complex with 3′-8-oxorG:A at the post-catalytic step, indicating the subsequent formation of a phosphodiester bond between the 3′-OH and 5′-PO_4_ ends of the nick (Fig. 1E). The 2Fo–Fc maps at 1.0σ demonstrate that the AMP moiety has continuous density for almost all the atoms in the LIG1 structures captured at the pre-catalytic step when AMP is bound to the 5′-PO_4_ end of the nick. The 2Fo–Fc map of the LIG1^EE/AA^/3′-8-oxorG:A structure captured at the post-catalytic step reveals a continuous density between the DNA ends of the nick (Supplementary Fig. 1). The map calculation included simulated annealing to remove bias.

**Table 1.** X-ray data collection and refinement statistics of LIG1 structures

	LIG1^EE/AA^3′-8oxorG:A(9YHU)	LIG1^EE/AA^3′-8oxorG:C(9YHV)	LIG1^EE/AA^3′-8oxodG:A(9YHW)	LIG1^EE/AA^3′-8oxodG:C(9YHX)	LIG1^WT^3′-8oxodG:C(9YHY)
Data collection					
Space group	P2_1_2_1_2_1_	P2_1_2_1_2_1_	P2_1_2_1_2_1_	P2_1_2_1_2_1_	P2_1_2_1_2_1_
Cell dimensions					
*a, b, c* (Å)	74.7 101.9 116.0	64.1 115.6 125.6	74.5 100.1 115.2	63.9 116.2 125.9	63.5 115.6 125.2
α, β, γ (°)	90	90	90	90	90
Resolution (Å)	25–1.96 (2.08–1.96)	37–2.80 (2.98–2.80)	30–2.56 (2.63–2.56)	25–2.96 (3.14–2.96)	25–2.76 (2.83–2.76)
*R*_meas_	0.04 (0.21)	0.05 (0.68)	0.17 (0.92)	0.11 (1.48)	0.08 (1.06)
*I* / σ (*I)*	21.12 (5.48)	16.61 (2.26)	15.95 (3.66)	13.69 (2.28)	16.01 (1.85)
CC_1/2_	0.97 (0.97)	0.99 (0.79)	0.99 (0.85)	0.99 (0.77)	0.99 (0.81)
Completeness (%)	99.0 (97.7)	99.5 (98.0)	99.8 (99.1)	99.5 (99.7)	99.7 (99.4)
Redundancy	3.5 (3.5)	3.1 (3.2)	13.5 (13.1)	7.1 (7.1)	6.9(6.6)
Wilson B-factor	36.04	75.97	42.98	60.90	74.66
Refinement					
Resolution (Å)	24.56–1.96	36.83–2.81	29.86–2.56	24.85–2.96	24.76–2.76
No. reflections	63 680	23 296	28 293	20 007	24 396
*R*_work_/*R*_free_	17.3/20.4	22.4/26.2	18.6/22.9	20.8/25.3	23.2/28.3
Non-H atoms:	5973	4993	5783	5299	5013
Protein	4786	4184	4810	4524	4230
DNA/RNA	737	736	736	734	734
AMP/ligand	22	22	42	22	22
H_2_O	428	52	195	19	30
Average B- factor (Å2):	32.75	91.67	39.05	80.37	99.42
Protein	32.46	96.22	39.49	83.98	103.43
DNA/RNA	32.92	66.46	36.71	58.36	77.16
AMP/ligand	25.44	80.96	44.54	79.96	95.84
H_2_O	36.15	79.20	35.94	71.29	81.45
R.M.S.D.					
Bond lengths (Å)	0.010	0.002	0.003	0.003	0.003
Bond angles (°)	1.107	0.515	0.548	0.561	0.640

Each data collected from single crystal.

Values in the parenthesis are highest-resolution shell.

The overlay of LIG1 pre- and post-catalytic structures shows a conformational change at the phosphate group of AMP before the formation of a phosphodiester bond, demonstrating how remodeling of ligase active site contacts propels the ligation reaction forward. This structural adjustment has also been previously reported for T4 RNA ligase 1 [49]. The superimposition of the overall LIG1 structures determined at the pre- and post-catalytic steps of the ligation reaction demonstrated differences in the distances relative to the 3′-OH and 5′-PO_4_ ends of the nick (Supplementary Fig. S2). We observed Watson–Crick base pairing for 3′-8-oxodG:C and 3′-8-oxorG:C and Hoogsteen base pairing for 3′-8-oxodG:A and 3′-8-oxorG:A (Supplementary Fig. S3). The root mean square deviation of the LIG1 structures was not >0.7 Å for any main-chain atoms, suggesting that they have similar conformations (Supplementary Table S7). Furthermore, in line with our previously solved LIG1 structures [45–47], we demonstrated that LIG1, which consists of oligonucleotide-binding, DNA-binding, and adenylation domains, completely envelopes the nick (Supplementary Fig. S4). We determined the structure of LIG1/nick complex with 3′-8-oxodG:C for both the wild type and the EE/AA mutant. The overlay of LIG1^WT^/3′-8-oxodG:C and LIG1^EE/AA^/3′-8-oxodG:C structures revealed no difference in the orientation of 3′-OH with respect to 5′-PO_4_ at the nick, which also confirms the presence of E346A and E592A amino acid substitutions at the high-fidelity site (Supplementary Fig. S5).

**Figure 2.**
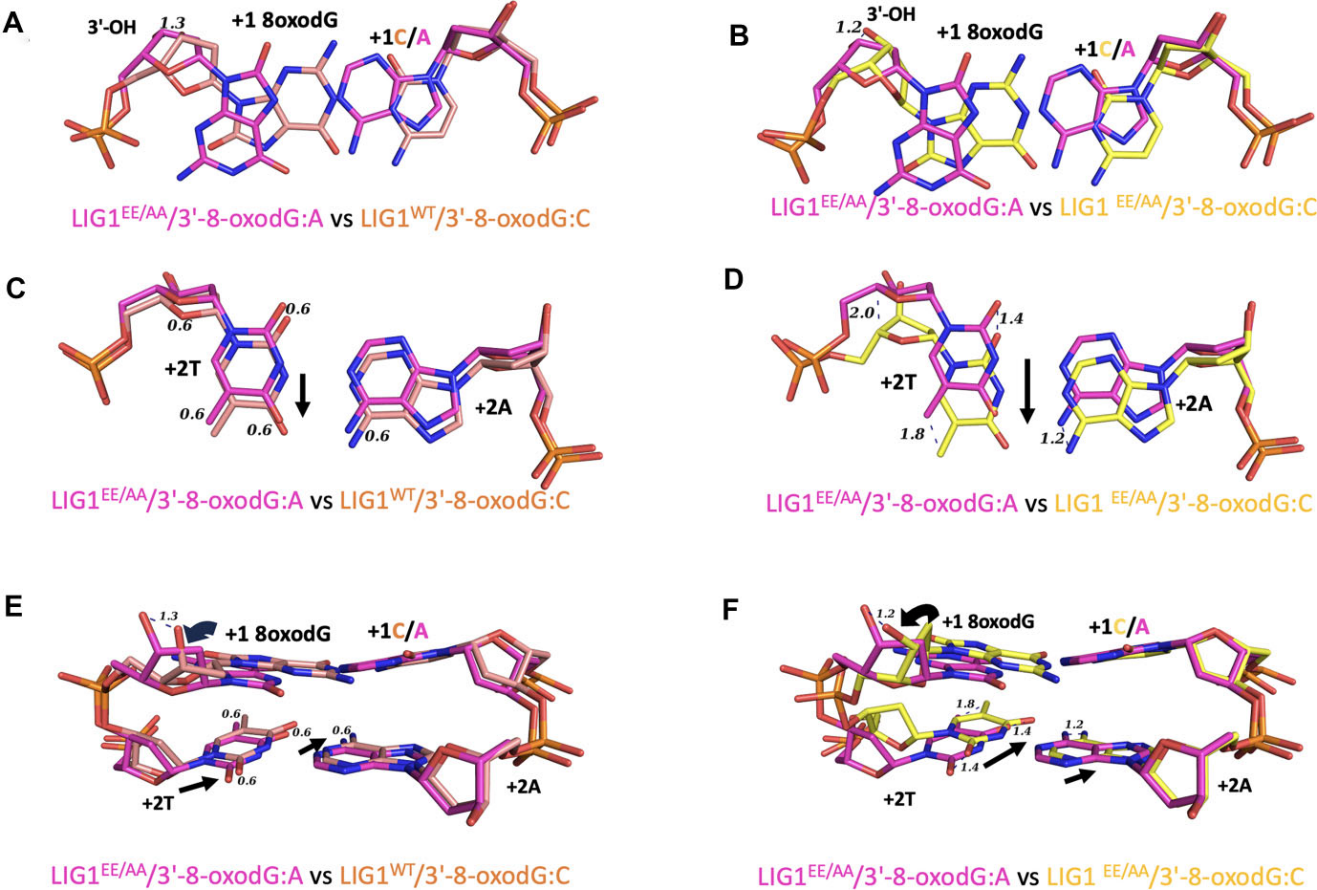
Superimposition of LIG1 structures demonstrates differences in the distances at nucleotides around nick site depending on the dual coding potential of the oxidative lesion. (**A, B**) Overlays of LIG1^WT^/3′-8-oxodG:C and LIG1^EE/AA^/3′-8-oxodG:A, LIG1^EE/AA^/3′-8-oxodG:C, and LIG1^EE/AA^/3′-8-oxodG:A structures show that the misalignment in the deoxyribose of 3′-8-oxoG. (**C, D**) The overlay of +2 nucleotides shows a movement of 0.6 Å and 1.2 Å at +2 nucleotides T:A of LIG1^WT^/3′-8-oxodG:C and LIG1^EE/AA^/3′-8-oxodG:C, respectively. (**E, F**) The overlay of +1 and +2 nucleotides of the nick shows overall conformational changes due to dual coding potential of 8-oxodGTP(*anti*):C(*anti*) and 8-oxodGTP(*syn*):A(*anti*), which forms non-mutagenic Watson–Crick and mutagenic Hoogsteen base pairing, respectively, at the ligase active site.

The superimposition of LIG1 structures in complex with nick DNA containing 3′-8-oxodG reveals a difference in the deoxyribose group of the oxidative lesion depending on the identity of the templating base (A or C) that forms Hoogsteen or Watson–Crick base pairing in -*syn* or -*anti* conformation, respectively (Fig. 2). Furthermore, the overlay of LIG1^WT^/3′-8-oxodG:C with LIG1^EE/AA^/3′-8-oxodG:A and LIG1^EE/AA^/3′-8-oxodG:C with LIG1^EE/AA^/3′-8-oxodG:A structures revealed a movement in the deoxyribose of the nick due to the Hoogsteen base pairing in the structure of LIG1^EE/AA^/3′-8-oxodG:A (Fig. 2A and B). We observed that LIG1^WT^/3′-8-oxodG:C and LIG1^EE/AA^/3′-8-oxodG:C moved by 1.3 Å and 1.2 Å, respectively, versus 3′-8-oxodG:A. The deoxyribose of LIG1^WT^/3′-8-oxodG:C and LIG1^EE/AA^/3′-8-oxodG:C moved +2T nucleotides by 0.6 Å and 1.8 Å, respectively, leading to shifts of 0.6 Å and 1.2 Å, respectively, at the +2A position of the template (Fig. 2C–F).

**Figure 3.**
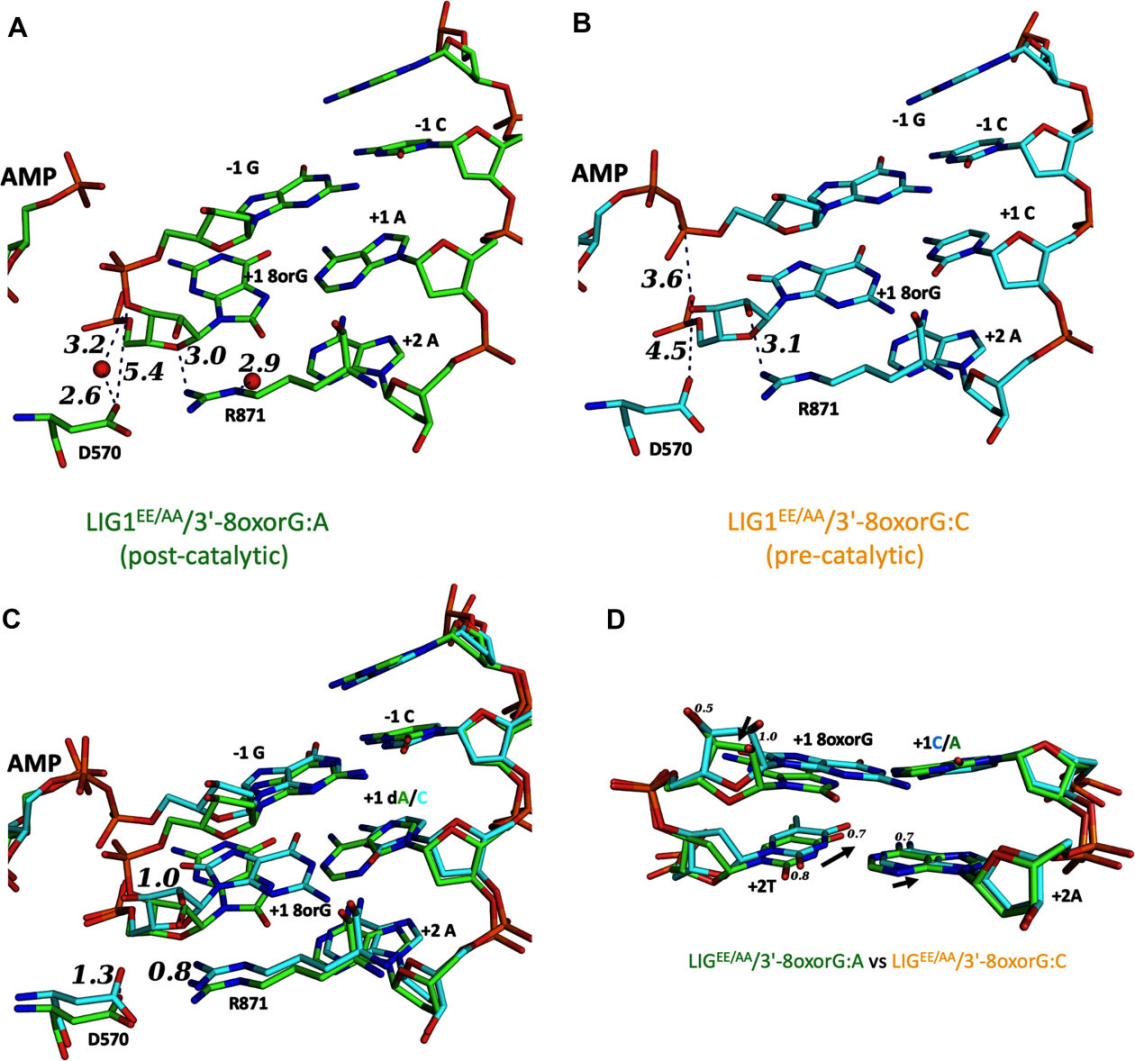
LIG1 structures of DNA–RNA heteroduplexes containing 8-oxorG. (**A**–**C**) The interaction network between 3′-8-oxorG and the active site amino acid residues D570 and R871. The overlays of LIG1^EE/AA^/3′-8-oxorG:A and LIG1^EE/AA^/3′-8-oxorG:C structures demonstrate that both complexes adopt similar conformations and interactions with the ligase active site residues D570 and R871 positional deviation of ∼ 1 Å. (**D**) The overlay of +1 and +2 nucleotides relative to nick site shows overall conformational changes due to Watson–Crick versus Hoogsteen base pairing between LIG1 structures.

LIG1^EE/AA^/3′-8-oxorG:A and LIG1^EE/AA^/3′-8-oxorG:C structures revealed that 8-oxorG moves 0.9 Å closer to the 5′-end of the nick, which reduces the distance between the DNA ends of 5′-PO_4_ and 3′-OH to 3.6 Å, and both LIG1 structures share a similar interaction network with the ligase active site residues D570 and R871 (Fig. 3A and B). The superimposition of LIG1^EE/AA^ structures for +1 of 3′-8-oxorG:C and +2A of 3′-8-oxorG:A shows that the ribose moiety of both +1 and +2 nucleotides moves 0.7 Å, which reflects a movement in the +2 template nucleotide (Fig. 3C and D). These conformational changes at the nick site could be the reason for the capture of LIG1 at the pre- or post-catalytic step of the ligation reaction in the presence of 3′-8-oxorG:C and 3′-8-oxorG:A, respectively.

### Ligation efficiency of nick DNA with oxidatively damaged ends by LIG1

**Figure 4.**
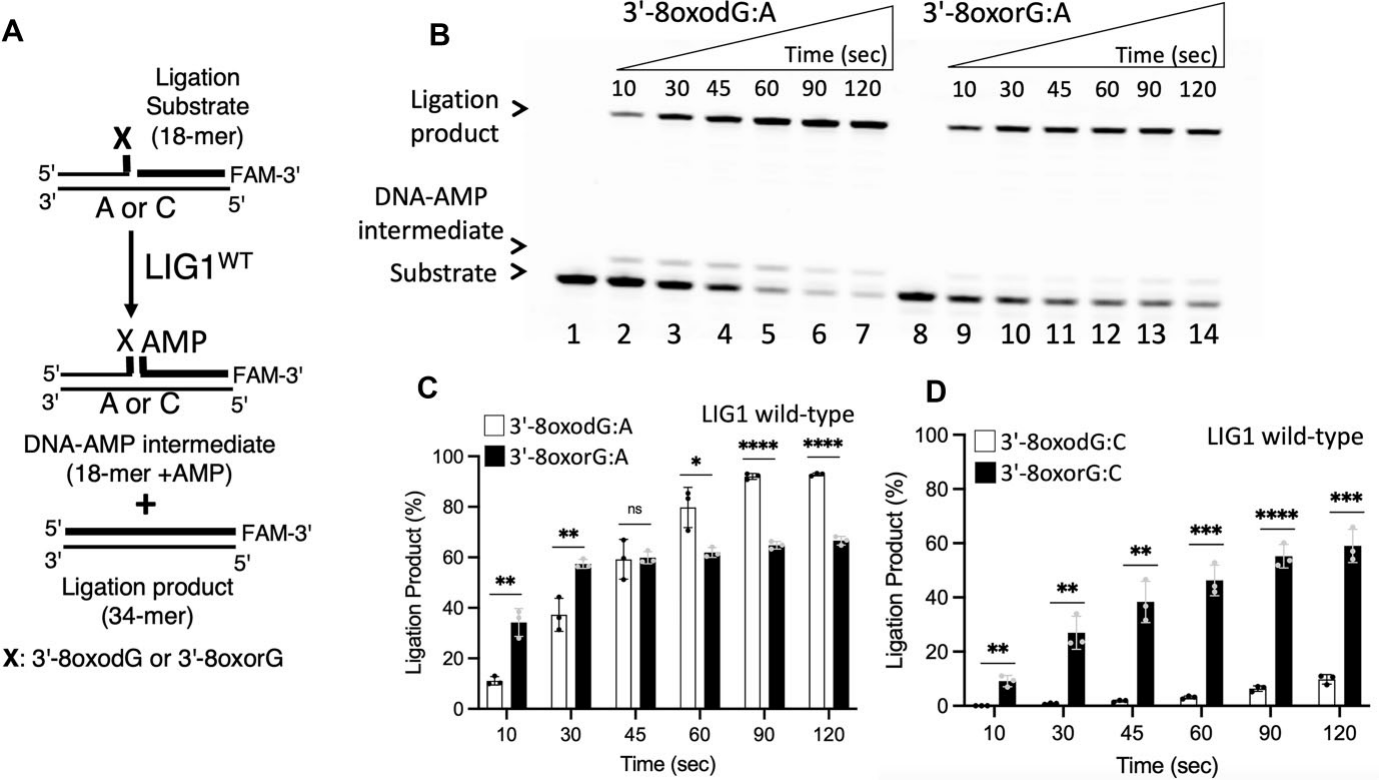
Ligation of the nick DNA substrates with oxidatively damaged ends by LIG1. (**A**) Scheme shows reaction substrate and products observed in the ligation assays in the presence of nick DNA substrates containing 3′-8-oxodG or 3′-8-oxorG opposite template A. (**B**) Lanes 1 and 8 are the negative enzyme controls of the nick DNA substrates containing 3′-8-oxodG:A and 3′-8-oxorG:A, respectively. Lanes 2–7 and 9–14 are the ligation products by LIG1 in the presence of nick DNA substrates containing 3′-8-oxodG:A and 3′-8-oxorG:A, respectively, and correspond to time points of 10, 30, 45, 60, 90, and 120 s. (**C, D**) Graphs show the time-dependent change in the amount of ligation products. Data points represent three independent replicates. Bar height is the mean, and error bars represent the SD. n.s., not significant; *P < .05 by ordinary two-way analysis of variance (ANOVA) with multiple comparisons.

We next investigated the ligation efficiency of LIG1 in the presence of nick DNA substrates containing 8-oxodG and 8-oxorG at the 3′-end opposite the template base A or C (Fig. 4A). Our results demonstrated mutagenic ligation of 3′-8-oxodG:A and 3′-8-oxorG:A by LIG1 (Fig. 4B). Similarly, we observed efficient ligation of nick DNA containing 3′-8-oxorG:C, whereas the ability of LIG1 to seal the nick DNA substrate with 3′-8-oxodG:C decreased (Supplementary Fig. S6A). We did not observe a significant difference in the amount of mutagenic ligation products for nick substrates containing 3′-8-oxodG and 3′-8-oxorG paired with template A (Fig. 4C). However, there was an ∼6-fold difference in the nick sealing products of 3′-8-oxodG and 3′-8-oxorG paired with template C (Fig. 4D). In terms of the identity of the template base, the comparison of the ligation efficiency revealed ∼100-fold difference for 3′-8-oxodG opposite the template base A over C and no difference for 3′-8-oxorG between templates A and C (Supplementary Fig. S7A and B).

**Figure 5.**
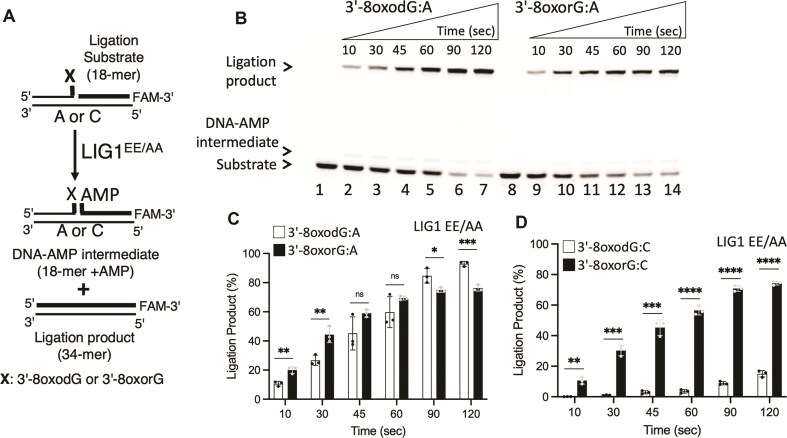
Ligation of the nick DNA substrates with oxidatively damaged ends by LIG1 low-fidelity mutant. (**A**) Scheme shows reaction substrate and products observed in the ligation assays in the presence of nick DNA substrates containing 3′-8-oxodG:A or 3′-8-oxorG:A. (**B**) Lanes 1 and 8 are the negative enzyme controls of the nick DNA substrates containing 3′-8-oxodG:A and 3′-8-oxorG:A, respectively. Lanes 2–7 and 9–14 are the ligation products by LIG1 EE/AA mutant in the presence of the nick DNA substrates containing 3′-8-oxodG:A and 3′-8-oxorG:A, respectively, and correspond to time points of 10, 30, 45, 60, 90, and 120 s. (**C, D**) Graphs show the time-dependent change in the amount of ligation products. Data points represent three independent replicates. Bar height is the mean, and error bars represent the SD. n.s., not significant; *P < .05 by ordinary two-way ANOVA with multiple comparisons.

**Figure 6.**
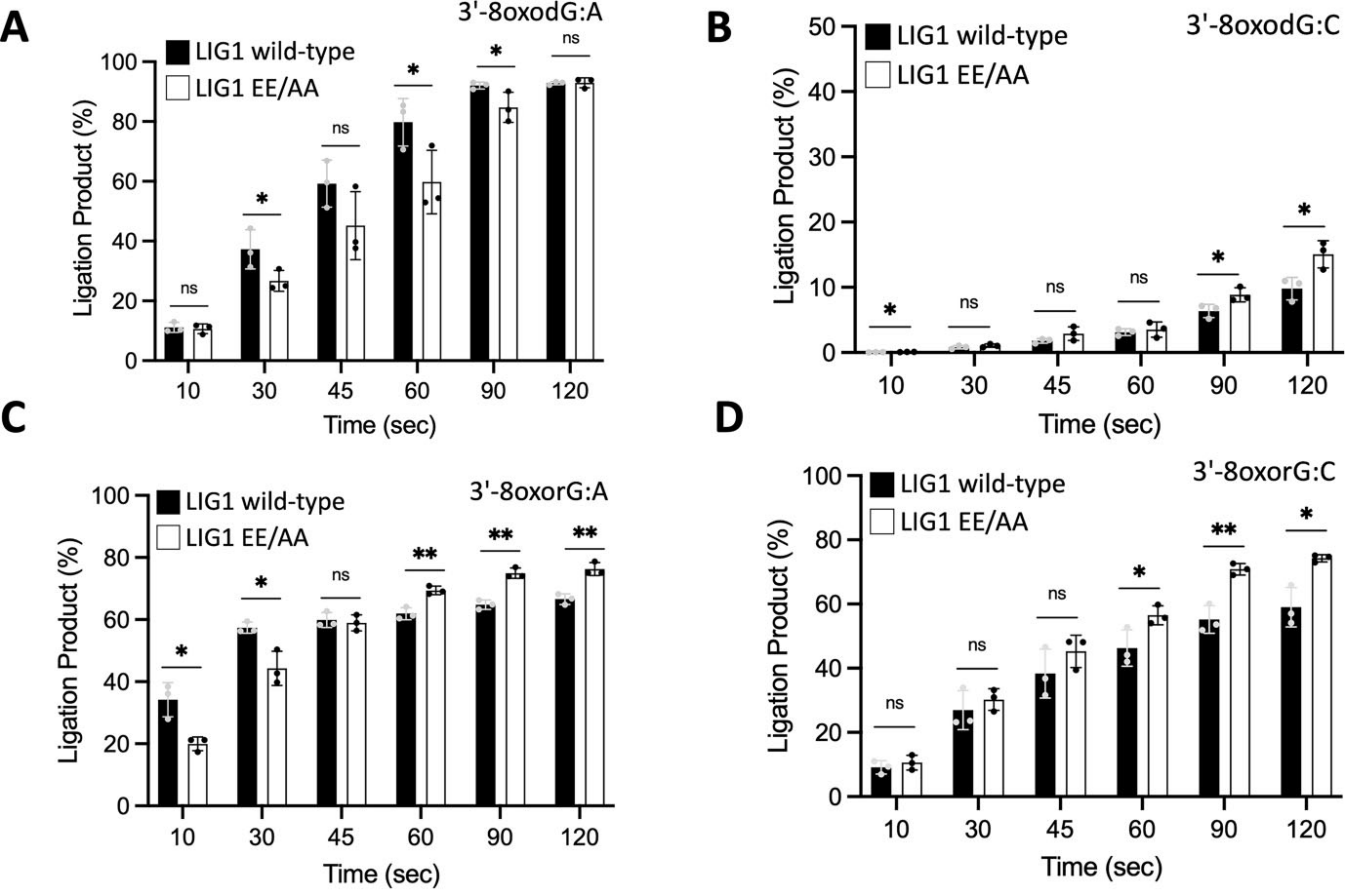
Comparison of ligation efficiency between LIG1 wild-type and EE/AA mutant. Graphs show the time-dependent change in the amount of ligation products by LIG1 wild-type and EE/AA mutant for the nick DNA substrates containing 3′-8-oxodG:A (**A**), 3′-8-oxodG:C (**B**), 3′-8-oxorG:A (**C**), and 3′-8-oxorG:C (**D**). Data points represent three independent replicates. Bar height is the mean, and error bars represent the SD. n.s., not significant; *P < .05 by ordinary two-way ANOVA with multiple comparisons.

We then compared the nick sealing efficiency of the LIG1^EE/AA^ low-fidelity mutant used for protein crystallization (Fig. 5A). Similar to the wild-type, our results demonstrated the mutagenic ligation of nicks containing 3′-8-oxodG:A and 3′-8-oxorG:A (Fig. 5B), and no significant difference in the amount of ligation product was detected (Fig. 5C). We also obtained relatively less efficient end joining by the LIG1^EE/AA^ mutant in the presence of the nick substrate containing 3′-8-oxodG:C and 3′-8-oxorG:C (Supplementary Fig. S6B); however, there was an ∼4-fold difference in the amount of nick sealing products (Fig. 5D). The comparison of ligation efficiency depending on the nature of the template base also revealed an ∼80-fold difference for 3′-8-oxodG opposite template base A over C and no difference between 3′-8-oxorG opposite A or C (Supplementary Fig. S7C and D). In control assays, we used the canonical nick substrate and obtained efficient ligation by both LIG1 proteins as expected (Supplementary Fig. S8). Overall, the comparison of LIG1 wild-type and the EE/AA mutant revealed no significant difference in the ligation efficiencies for nick DNA substrates that we solved the crystal structures (Fig. 6). For both LIG1 proteins, we observed mutagenic ligation of 3′-8-oxorG or 3′-8-oxodG when base paired with A and diminished nick sealing of 3′-8-oxodG when base paired with C (Fig. 6A–C), while the presence of 3′-8-oxorG templating C at the nick can be efficiently joined (Fig. 6D).

In our recent report [46], we demonstrated that LIG1 can ligate the nick DNA substrate with a single ribonucleotide at the 3′-end (rA:T and rG:C) as efficiently as Watson–Crick base-paired ends (3′-dA:T and 3′-dG:C). Furthermore, the structure of the LIG1^EE/AA^/3′-RNA–DNA heteroduplex was determined at the post-catalytic step of the ligation reaction, demonstrating a lack of sugar discrimination [46]. In the present study, to understand the impact of oxidatively damaged ends on nick sealing in the presence of a ribonucleotide, we next compared ligation efficiency of the nick substrates containing 3′-rG:C versus 3′-8-oxorG:C. Our results revealed more efficient ligation in the presence of a nick substrate containing 3′-rG:C (Supplementary Fig. S9). The overlay of the LIG1^EE/AA^/3′-rG:C and LIG1^EE/AA^/3′-8-oxorG:C structures revealed that 3′-8-oxorG moves ∼1 Å closer to the 5′-PO_4_ of the nick, leading to 3′-OH away from the ligase active site residue D570 but maintaining the same orientation (Supplementary Fig. S10), which could explain the similar ligation efficiency between nick substrates with 3′-rG:C and 3′-8oxorG:C.

### Interplay between APE1 and LIG1 during processing of nicks with oxidatively damaged ends

It has been demonstrated that APE1 proofreads mis-insertion errors introduced by DNA polymerase β during base excision repair [48]. Similarly, the studies using human cell extracts *in vitro* demonstrated that the repair of 3′-8-oxoG is resistant to excision by DNA glycosylases involved in the repair of oxidative lesions and that APE1 can remove 3′-8-oxoG lesions when incorporated by DNA polymerases during repair [50]. MTH1, an oxidized nucleotide pool sanitizer, exerts weak activity on 8-oxorGTP or 8-oxorG, making it uncertain whether the reduced activity is sufficient to protect the cell from oxidized ribonucleotides [51, 52]. We previously reported that APE1 can physically interact with both LIG1 and LIG3α [53].

In the present study, we investigated the role of APE1 in proofreading nick substrates containing 3′-8-oxodG:A, 3′-8-oxodG:C, 3′-8-oxorG:A, and 3′-8-oxorG:C. Our results revealed a time-dependent increase in the removal of 8-oxorG templating A by APE1 and its coordination with LIG1 for proofreading coupled with nick sealing (Supplementary Fig. S11). We observed that the products of APE1 exonuclease removal are mainly converted by ligation of the resulting gap product by LIG1, demonstrating a functional coordination during the processing of nicks with damaged ends. Our results demonstrated that APE1 activity for the removal of 8-oxorG templating C and 8-oxodG templating A or C was relatively weak, although LIG1 can still attempt to seal gaps or nick DNA intermediates at different efficiencies depending on the architecture of the 3′-terminus:template base (Supplementary Figs. 12-14). These findings could contribute to our understanding of how a multiprotein complex (APE1 and LIG1) ensures accuracy at the final steps during damage repair.

## Discussion

Oxidative DNA damage plays a role in various diseases and contributes to the spontaneous mutations implicated in cancer and aging [1–3]. Among the nucleotides in the cellular dNTP and rNTP pools, dGTP and rGTP are the most prone to oxidation because of the high redox potential of guanines, and their oxidation results in the formation of 8-oxo-7,8-dihydro-2-deoxyguanosine triphosphate (8-oxodGTP) and 8-oxo-guanosine-5′-triphosphate (8-oxorGTP) [7–9]. Despite the expression of MTH1, which sanitizes oxidized nucleotides, very small amounts of 8-oxodGTP (<1% of the total dGTP pool as shown in mitochondria) would be sufficient to promote mutagenesis and drive cancer progression [11, 26, 31]. Several DNA polymerases mis-incorporate oxidized nucleotides in either -*anti* or -*syn* conformation opposite to either C or A, respectively, leading to A–C or G–T transversion mutations following replication if left unrepaired [10, 27]. Furthermore, high levels of oxidatively damaged RNA are closely linked to aging and neurodegenerative diseases [13].

The complex network of DNA repair pathways is finalized by two consecutive steps involving nucleotide incorporation by a DNA polymerase and the subsequent joining of the resulting nick repair product in the DNA backbone by DNA ligase as an ultimate ligation step [37]. Upon incorporation of the oxidized nucleotides (8-oxodGTP or 8-oxorGTP) by DNA polymerases with no proofreading capacity, the resulting insertion product is the nick with 3′-8oxodG or 3′-8oxorG, which could compromise the ability of DNA ligase to seal DNA ends; therefore, ligation efficiency would be interrupted at the final step of DNA repair pathways [38, 39].

In the present study, we characterized how LIG1 engages with potentially mutagenic repair intermediates containing oxidatively damaged ends, and our structures provided an atomic insight into the strategies that the ligase active site utilizes to discriminate 8-oxoG at the 3′-end of a nick depending on the dual coding potential of the oxidative lesion. We captured LIG1 engaging with 3′-8-oxorG:A at the post-catalytic step, while the ligase/nick complex structures of 3′-8-oxodG:A, 3′-8-oxodG:C, and 3′-8-oxorG:C were determined at the pre-catalytic step of the ligation reaction.

It has been previously reported that LIG1 fidelity is mediated by catalytic Mg^2+^-dependent DNA binding that the enzyme employs during the adenyl transfer and nick sealing steps of the ligation reaction, and the ligase fidelity is governed by a high-fidelity interface between LIG1, Mg^2+^, and the DNA substrate [44]. Furthermore, the mutations at the conserved glutamate residues, E346A and E592A, lead to lower LIG1 fidelity (referred to as EE/AA) and create an open cavity that enables the ligase active site to accommodate modified ends at the nick site. Accordingly, we utilized this low-fidelity mutant (LIG1^EE/AA^) in our previously reported LIG1 structures in complex with a nick containing mismatches (G:T and A:C) or ribonucleotides (rA:T and rG:C) at the 3′-end [45–47]. In the present study, we further utilized the LIG1^EE/AA^ mutant to crystallize the protein in complex with nick DNA substrates harboring 3′-8oxodG:A, 3′-8oxodG:C, 3′-8oxorG:A, and 3′-8oxorG:C. In addition, we used the LIG1 wild-type (LIG1^WT^) for crystallization with nick DNA containing 3′-8oxodG:C.

We demonstrated no significant difference in the ligation efficiency between LIG1 wild-type and the EE/AA mutant for any of the nick substrates (3′-8-oxodG:A or C and 3′-8-oxorG:A or C) in the ligation assays that included Mg^2+^ (Fig. 6). Similarly, our real-time nick DNA binding measurements revealed similar nick DNA binding modes for LIG1 wild-type and the EE/AA mutant (Supplementary Fig. S15). However, it is important to note that we pre-treated the LIG1^EE/AA^ protein with EDTA during its purification and then crystallized the mutant in complex with nick substrates in the absence of Mg^2+^. Interestingly, we captured only the structure of LIG1^EE/AA^/3′-8-oxorG:A at the post-catalytic step of the ligation reaction, during which a final phosphodiester bond between 3′-OH and 5′-PO_4_ ends at the nick is formed (Fig. 1). To further explore the ligation efficiency under crystallization conditions, we performed ligation assays in the absence of Mg^2+^ using LIG1^EE/AA^ mutant that was purified with EDTA pre-treatment. Our results revealed the ligation products only for the nick substrate containing 3′-8-oxorG:A (Supplementary Figs. 16 and 17). In addition to our structural observations (Figs. 1 –3), these results could explain why the LIG1/3′-8oxorG:A structure is captured at the post-catalytic step when the ligation reaction progresses, with the final formation of a phosphodiester bond between DNA ends at the nick.

Similarly, we previously solved the LIG^EE/AA^ structures at the post-catalytic step in the presence of 3′-rA:T and 3′-rG:C [46]. Accordingly, we suggest a mechanism (Supplementary Scheme 1) through which a phosphodiester bond can form through a condensation reaction between DNA ends: this involves a water bridge between the ligase active site residues D570 and the 3′-OH, along with the interaction between the 2′-OH of the ribose and R871 side chain. This interaction network could establish a chemically favorable conformation. The interaction with R871 causes electron localization at the 2′-C, promoting the formation of a π-bond between the 2′ and 3′ carbons of −1 rA/G at the nick. Moreover, a water molecule positioned between the 3′-OH of +1 rA/rG/8-oxorG and D570 facilitates the departure of a water leaving group from the 3′-carbon of +1 rA/rG/8-oxorG, generating a carbocation at the 3′-end. This sets the stage for a nucleophilic attack by the -OH group of the 5′-PO_4_ of +1G at the nick, leading to the formation of a phosphate linkage between the 5′-PO_4_ of +1G and the 3′-C of +1 rA/rG/8-oxorG through an oxygen bridge.

In the ligation assays, we showed the mutagenic ligation of nick DNA substrates with 3′-8-oxodG:A and 3′-8-oxorG:A and an efficient nick sealing of 3′-8-oxorG:C, while the end joining of 3′-8-oxodG:C was greatly compromised by LIG1 (Fig. 6). The overlay of LIG1 structures for nicks containing 3′-8-oxodG:A versus 3′-8-oxodG:C demonstrated a conformational change in the deoxyribose group of 8-oxodG and movement at +2T and template +2A nucleotides relative to the 3′-OH strand of the nick (Fig. 2). This could explain the relatively less efficient end joining of nicks with 3′-8-oxodG:C. The superimposition of LIG1^EE/AA^/nick complexes containing oxidatively damaged ends with our previously solved structure of LIG1^EE/AA^/3′-dG:C provides further insight into this movement (Supplementary Fig. S18). In particular, we did not observe a conformational change at the +2 nucleotide, which has no effect on the overall movement at 3′-8-oxodG:A. However, the overlay of LIG1 structures for nicks containing 3′-dG:C versus 3′-8-oxodG:C shows a movement at +2 nucleotides relative to the 3′-OH strand of the nick (1.4 Å and 2.0 Å at +2A and +2T, respectively), which causes a significant displacement in the opposite direction to the active site. Furthermore, 8-oxodG:A base pairing in -*syn* conformation provides more flexibility at 3′-8-oxodG toward the 5′-PO_4_ of the nick, whereas 8-oxodG:C base pairing in -*anti* conformation restrains the movement of 3′-8-oxodG toward the 5′-PO_4_ of the nick.

Although we observed efficient ligation of nick DNA substrates containing 3′-8-oxorG templating either A or C (Fig. 1), the overlay of LIG1^EE/AA^ structures of 3′-8-oxorG:A versus 3′-8-oxorG:C shows significant displacement (0.8 Å) at 3′-8-oxorG toward the 5′-end of the nick in LIG1^EE/AA^/3′-8-oxorG:C (pre-catalytic) with reference to LIG1^EE/AA^/3′-8-oxorG:A (post-catalytic) and highlights the differences in the interaction network between the 2′-OH of 3′-8-oxorG and the side chain R871 (Fig. 3A–C). This causes conformational changes at the nick and could explain the capture of the LIG1^EE/AA^ structures of 3′-8-oxorG:A and 3′-8-oxorG:C at different steps of the ligation reaction.

Overall, our findings provide biochemical and structural insights into the ligase/nick DNA interactions with mutagenic repair intermediates that could be formed because of DNA polymerase-mediated incorporation of oxidized nucleotides, which is critical given their roles in mutagenesis, cancer therapeutics, and bacterial antibiotics. Our LIG1 structures contribute to the understanding of how the ligase active site processes nicks depending on the dual coding potential of 8-oxodGTP(*anti*):C and 8-oxodGTP(*syn*):A while discriminating “wrong” sugars at the nick site. Understanding the molecular determinants that dictate ligase fidelity at the final step of DNA replication and repair pathways could reveal breakthrough platforms to manipulate the oxidative DNA damage response for future therapeutic targeting in cancer cells with higher levels of genotoxic stress and the development of more effective and selective DNA ligase inhibitors [54, 55].

## Supplementary Material

gkaf1344_Supplemental_File

## Data Availability

Atomic coordinates and structure factors for the reported crystal structures of LIG1 have been deposited in the RCSB Protein Data Bank under accession numbers for LIG1^EE/AA^ 3′-8-oxodG:A (9YHW), LIG1^EE/AA^ 3′-8-oxodG:C (9YHX), LIG1^WT^ 3′-8-oxodG:C (9YHY), LIG1^EE/AA^ 3′-8-oxorG:A (9YHU), and LIG1^EE/AA^ 3′-8-oxorG:C (9YHV). All data are contained within the manuscript. Further information and requests of materials used in this research are available from the authors upon reasonable request.

## Notes

Present address: Eppley Institute for Research in Cancer, Fred & Pamela Buffett Cancer Center, University of Nebraska Medical Center, Omaha, NE 68198, United States
