# Supplementary material for: Processing of DNA single-strand breaks with oxidatively damaged ends by LIG1
Source: Nucleic Acids Res. 2025 Dec 10;53(22):gkaf1344. doi: 10.1093/nar/gkaf1344 (PMC12693511; doi:10.1093/nar/gkaf1344)
Supplement: gkaf1344_Supplemental_File [file gkaf1344_supplemental_file.pdf]

Processing of DNA single-strand strand breaks with oxidatively damaged ends by LIG1

Kanal E. Balu[1], Danah Almohdar[1], Camden Lerner[1], Jacob Ratcliffe[1], Qun Tang[1], Tanay Parwal[1], Kar M. Lee[1], Aishwarya Prakash[2] and Melike Çağlayan[1*#]

[1] University of Florida, Gainesville, FL 32610

[2] University of South Alabama, Mitchell Cancer Institute, Department of Biochemistry and Molecular Biology, Mobile, AL 36604

[*]To whom correspondence should be addressed. E-mail: caglayanm@ufl.edu

[#]Present address: Eppley Institute for Research in Cancer, Fred & Pamela Buffett Cancer Center, University of Nebraska Medical Center, Omaha, NE 68198 E-mail: mcaglayan@unmc.edu

Supplementary Figures 1-18

Supplementary Tables 1-7

Supplementary Scheme 1

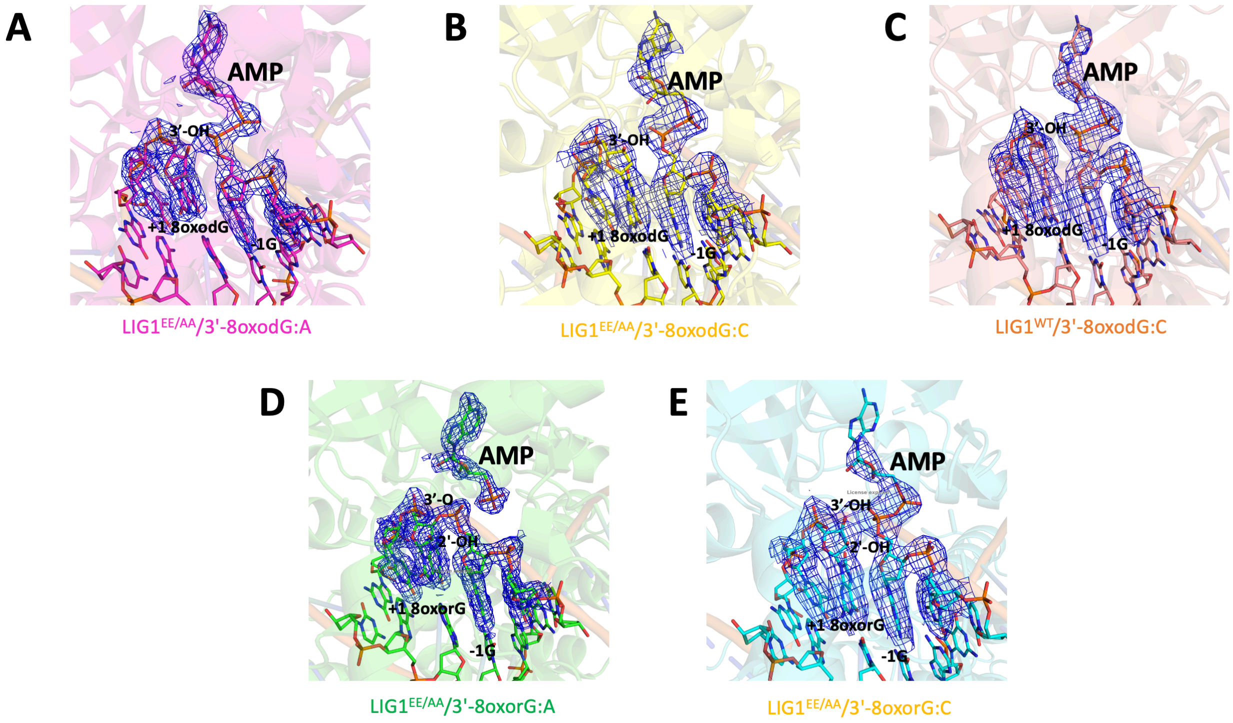

Supplementary Figure 1. Structures of LIG1/nick DNA complexes show differences in the positions of AMP. (A-E) Structures of LIG1$^{EE/AA}$ in complex with nicks containing 3'-8-oxodG:A, 3'-8-oxodG:C, 3'-8-oxorG:C and structure of LIG1^{WT} in complex with nicks containing 3'-8-oxodG:C were determined at the pre-catalytic step of the ligation reaction where AMP is bound to 5'-PO_4 end of nick. Structure of LIG1$^{EE/AA}$ in complex with nick containing 3'-8-oxorG:A was determined at the post-catalytic step of the ligation reaction where the map for AMP is incomplete. Final sigma weighted 2Fo-Fc mapped at 1.5 around AMP/nick site. The map calculation included simulated annealing to remove bias.

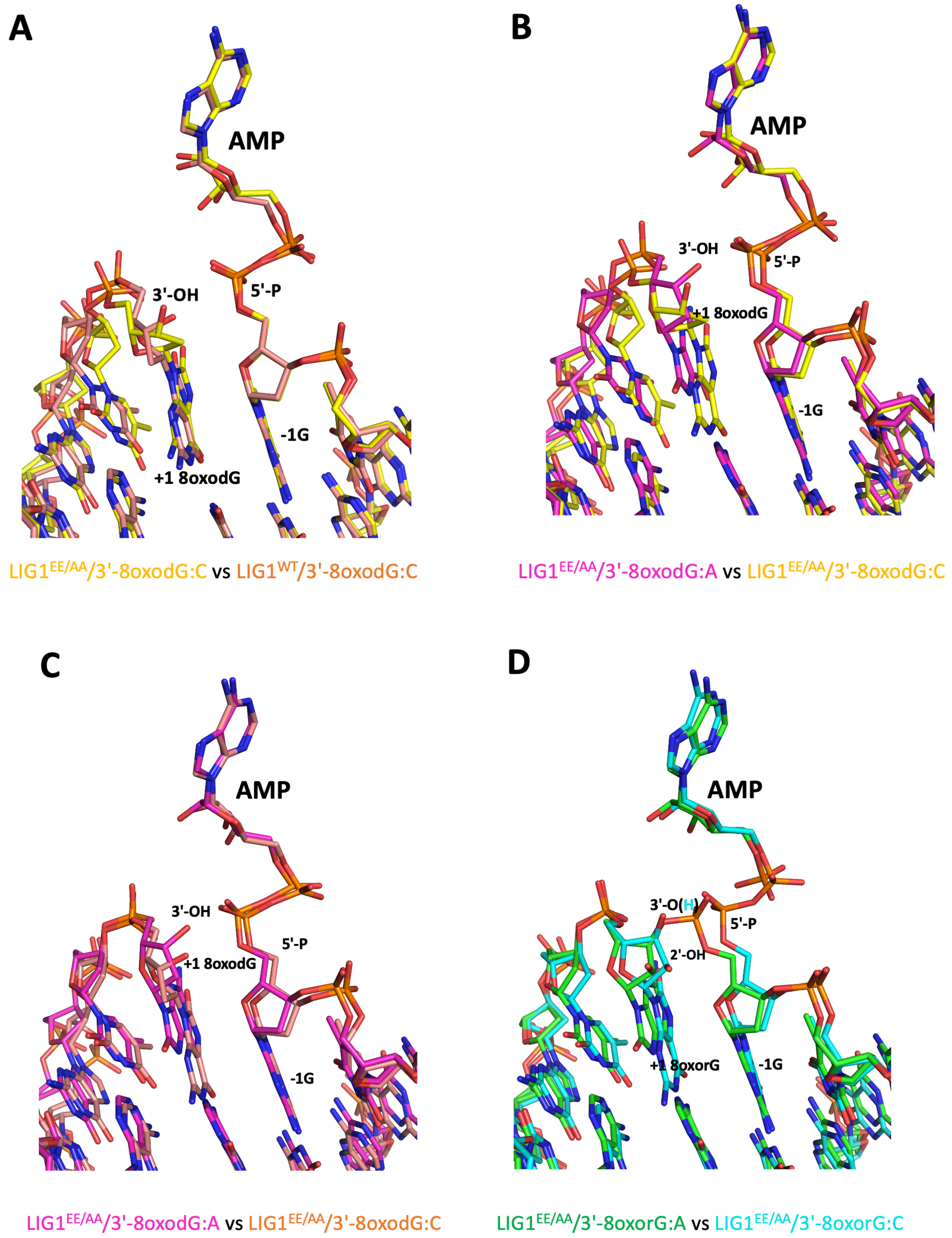
A
AMP
3'-OH
5'-P
-1G
+1 8oxodG
LIG1$^{EE/AA}$/3'-8oxodG:C vs LIG1^{WT}/3'-8oxodG:C
B
AMP
3'-OH
5'-P
+1 8oxodG
-1G
LIG1$^{EE/AA}$/3'-8oxodG:A vs LIG1$^{EE/AA}$/3'-8oxodG:C
C
AMP
3'-OH
+1 8oxodG
5'-P
-1G
LIG1$^{EE/AA}$/3'-8oxodG:A vs LIG1$^{EE/AA}$/3'-8oxodG:C
D
AMP
3'-O(H)
5'-P
2'-OH
+1 8oxorG
-1G
LIG1$^{EE/AA}$/3'-8oxorG:A vs LIG1$^{EE/AA}$/3'-8oxorG:C

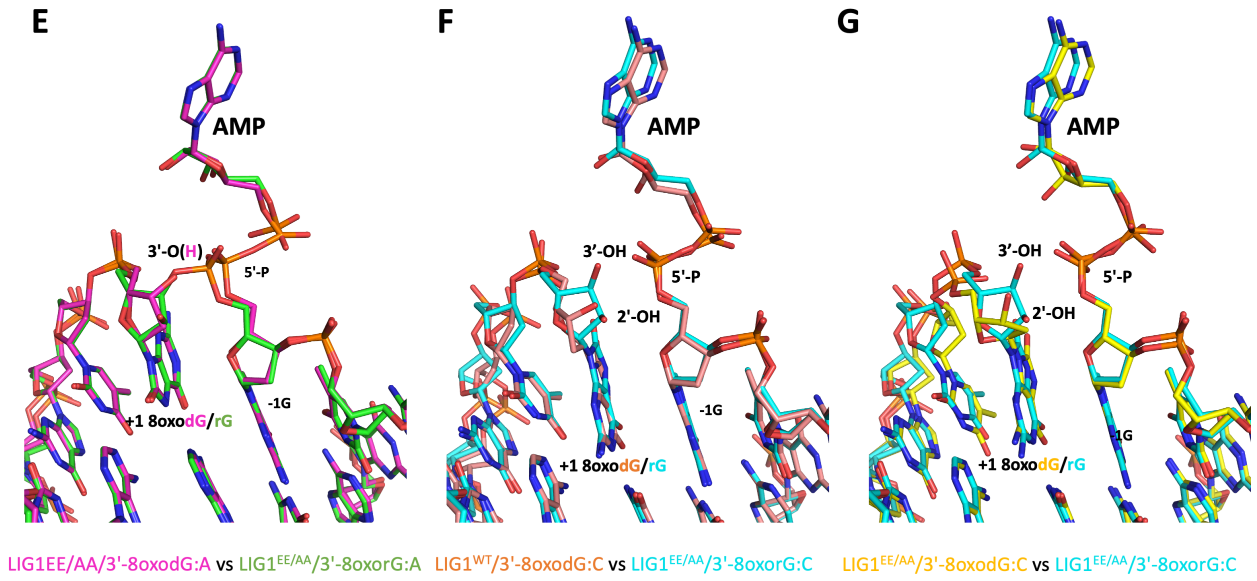

Supplementary Figure 2. Overlays of LIG1 structures containing oxidatively damaged ends. **(A)** Overlay of LIG1^{WT}/3'-8-oxodG:C and LIG1$^{EE/AA}$/3'-8-oxodG:C structures shows that 3'-OH of the nick shares the same position. (**B-C**) Overlay of LIG$^{EE/AA}$/3'-8-oxodG:A and LIG1$^{EE/AA}$/3'-8-oxodG:C structures and the overlay of LIG1$^{EE/AA}$/3'-8-oxodG:A and LIG1^{WT}/3'-8-oxodG:C structures show that 3'-OH of LIG$^{EE/AA}$/3'-8-oxodG:A of the nick moves closer to 5'-PO_4. **(D)** Overlay of LIG1$^{EE/AA}$/3'-8-oxorG:A and LIG1$^{EE/AA}$/3'-8-oxorG:C structures shows that the ribose sugar at the 3'-end of the nick shares similar conformation, yet it moves closer toward the 5'-end in the structure LIG1$^{EE/AA}$/3'-8-oxorG:A at post-catalytic step. **(E)** Overlay of LIG1$^{EE/AA}$/3'-8-oxodG:A and LIG1$^{EE/AA}$/3'-8-oxorG:A structures shows that 3'-OH of the nick shares same positions. (**F-G**) Overlay of LIG1^{WT}/3'-8-oxodG:C and LIG1$^{EE/AA}$/3'-8-oxorG:C structures and the overlay of LIG1$^{EE/AA}$/3'-8-oxodG:C and LIG1$^{EE/AA}$/3'-8-oxorG:C structures show that 3'-8-oxoG adopts similar orientation and conformational changes at the 3'-OH of nick.

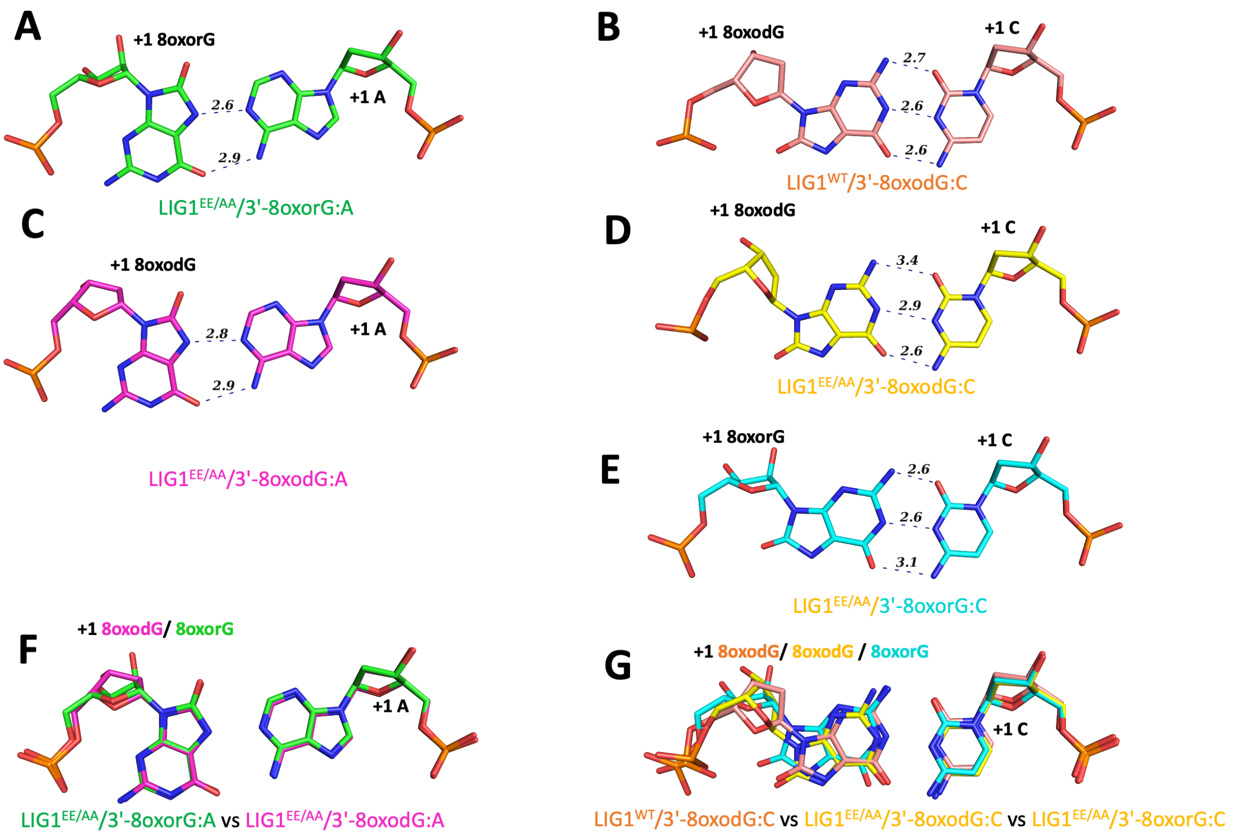

Supplementary Figure 3. Base pairing architecture of LIG1 structures. (A-E) Hoogsteen and Watson-Crick base pairing are shown for the structures of LIG1$^{EE/AA}$/3'-8-oxodG:A, LIG1$^{EE/AA}$/3'-8-oxorG:A, LIG1^{WT}/3'-8-oxodG:C, LIG1$^{EE/AA}$/3'-8-oxodG:C, and LIG1$^{EE/AA}$/3'-8-oxorG:C. **(F-G)** Overlays of LIG1 structures showing Hoogsteen base pairing (LIG1$^{EE/AA}$/3'-8-oxodG:A and LIG1$^{EE/AA}$/3'-8-oxorG:A) and Watson-Crick base pairing (LIG1^{WT}/3'-8-oxodG:C, LIG1$^{EE/AA}$/3'-8-oxodG:C and LIG1$^{EE/AA}$/3'-8-oxorG:C) show no difference in the base pairing architecture.

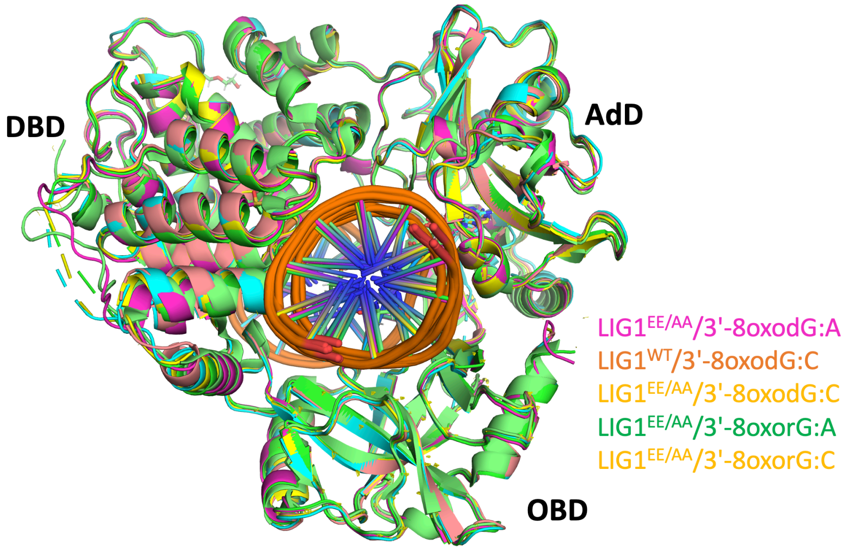

Supplementary Figure 4. Protein domain organization of LIG1/nick DNA complex structures. Superimposition of all LIG1 structures demonstrates a global conformation that the catalytic core, consisting of Adenylation (AdD), DNA-binding (DBD), and Oligonucleotide-binding (OBD) domains, encircles a nick containing oxidatively damaged ends.

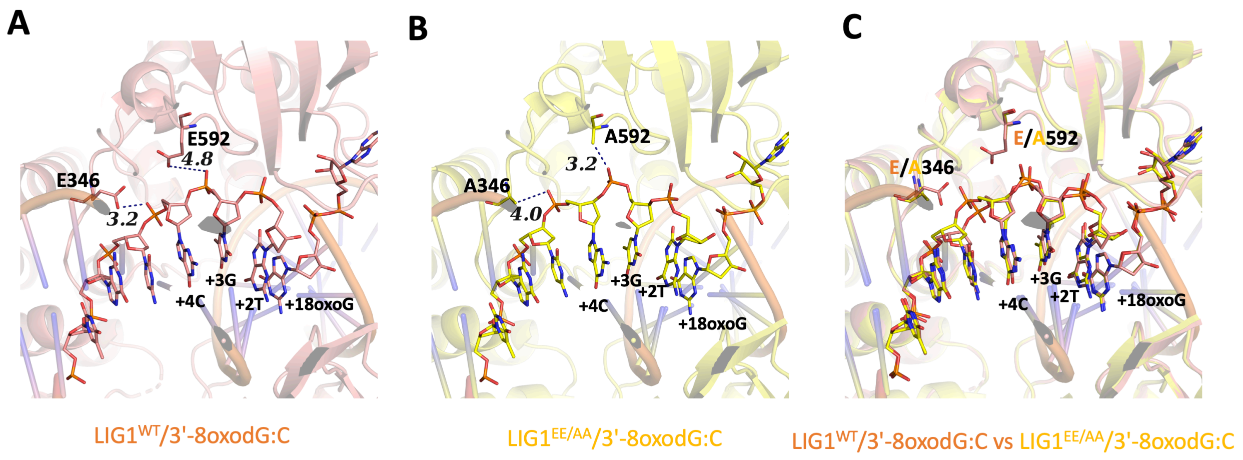

Supplementary Figure 5. LIG1 structures in the presence and absence of the mutation at the high-fidelity site. (A-B) Individual structures for $LIG1^{WT}$/3'-8-oxodG:C and $LIG1^{EE/AA}$/3'-8-oxodG:C show the positions of the high-fidelity site for LIG1 wild-type (E346 and E592) and EE/AA mutant (A346 and A592). **(C)** Overlay of both structures demonstrates no difference in the orientation of 3'-OH towards 5'-PO_4 at nick.

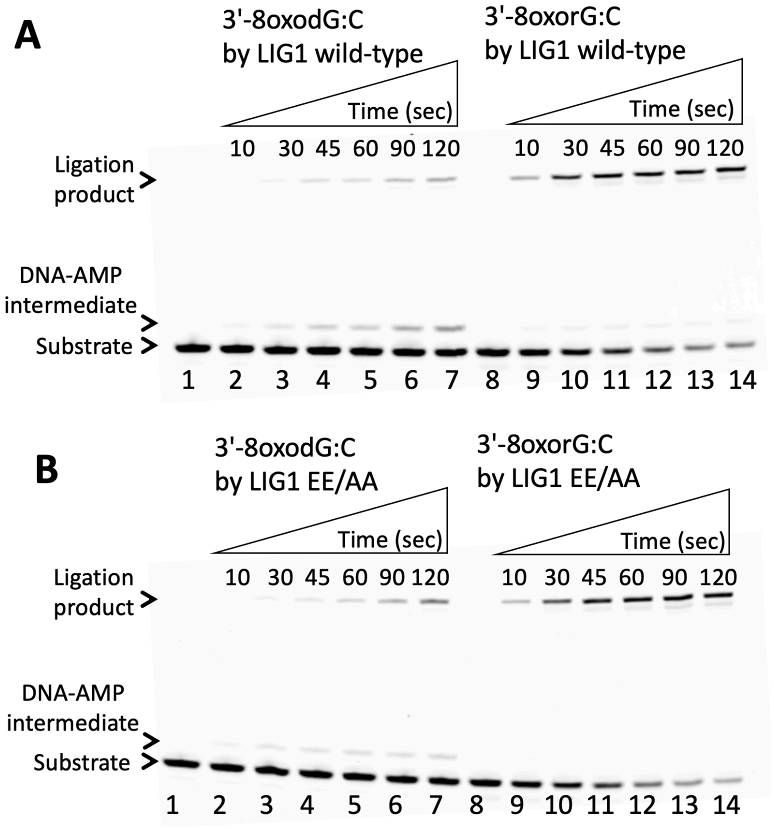

Supplementary Figure 6. Ligation of nick DNA substrates with oxidatively damaged ends by LIG1. (A-B) Lanes 1 and 8 are the negative enzyme controls of the nick DNA substrates containing 3'-8-oxodG:C and 3'-8-oxorG:C, respectively. Lanes 2-7 and 9-14 are the ligation products by LIG1 wild-type (A) and EE/AA mutant (B) for the nick DNA substrates containing 3'-8-oxodG:C and 3'-8-oxorG:C, respectively, and correspond to time points of 10, 30, 45, 60, 90, 120 sec. Bar graphs showing time-dependent formation of ligation products for both LIG1 proteins are presented in Figures 4-6.

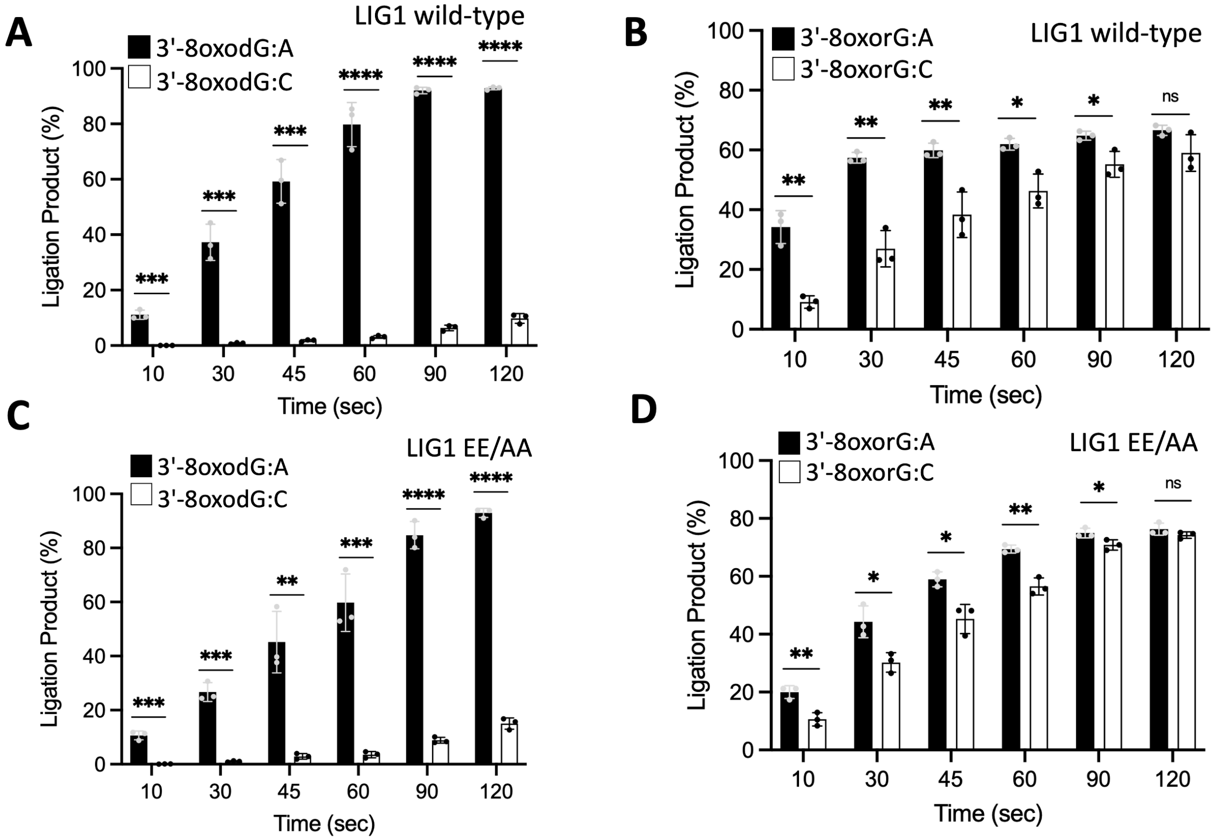

Supplementary Figure 7. Comparison of ligation efficiency for nick DNA substrates containing oxidatively damaged ends by LIG1 wild-type *versus* EE/AA mutant in the absence and presence of 3'-ribonucleotide. (A-D) Graphs show the time-dependent change in the amount of ligation products for nick DNA substrates containing 3'-8-oxodG or 3'-8-oxorG templating A or C by LIG1 wild-type (A-B) and EE/AA mutant (C-D). Data points represent three independent replicates. Bar height is the mean, and error bars represent the SD. n.s., not significant; *P < 0.05 by ordinary two-way ANOVA with multiple comparisons.

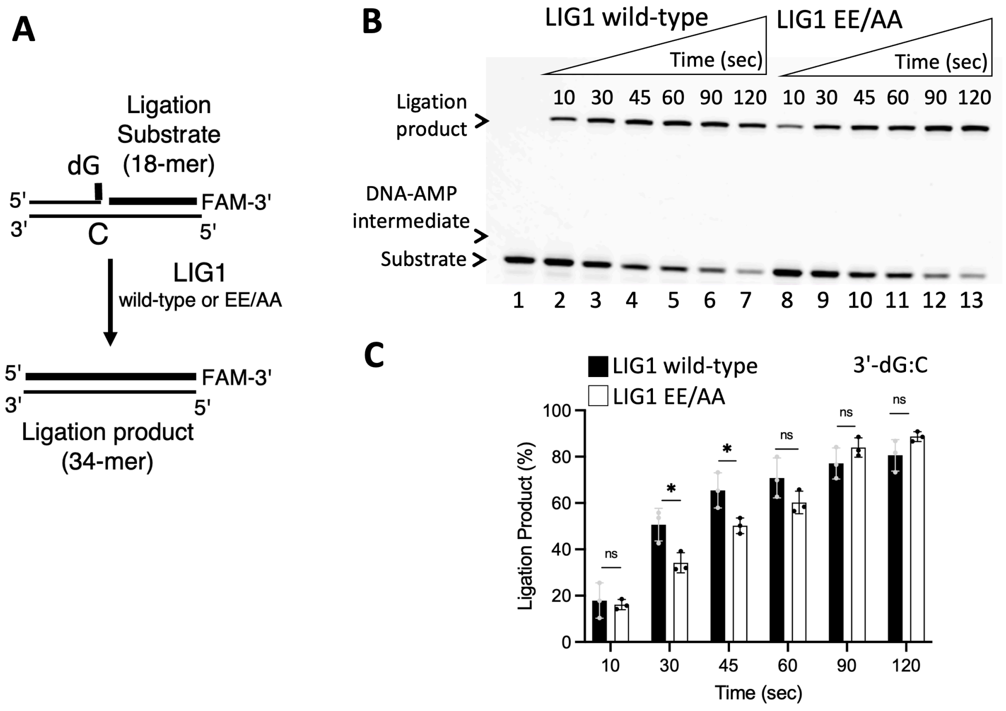

Supplementary Figure 8. Ligation of nick DNA substrate with canonical end by LIG1. (A) Scheme shows reaction substrate and product observed in the ligation assays in the presence of the nick DNA substrate containing 3'-dG:C. **(B)** Line 1 is the negative enzyme control of nick DNA substrate with 3'-dG:C. Lanes 2-7 and 8-13 are the ligation products by LIG1 wild-type and EE/AA mutant, respectively, and correspond to time points of 10, 30, 45, 60, 90, 120 sec. **(C)** Graph shows the time-dependent change in the amount of ligation products. Data points represent three independent replicates. Bar height is the mean, and error bars represent the SD. n.s., not significant; *$P < 0.05$ by ordinary two-way ANOVA with multiple comparisons.

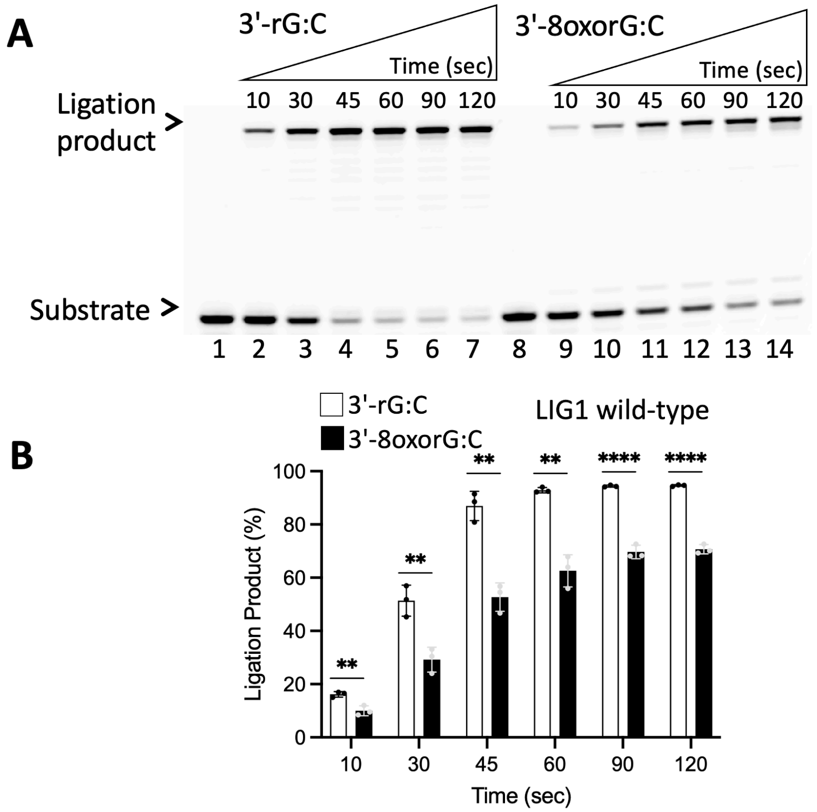

Supplementary Figure 9. Ligation of nick DNA substrates with undamaged *versus* damaged ribonucleotide-containing ends by LIG1. (A) Lanes 1 and 8 are the negative enzyme controls of nick DNA substrates containing 3'-rG:C and 3'-8-oxorG:C, respectively. Lanes 2-7 and 9-14 are the ligation products by LIG1 in the presence of nick DNA substrates containing 3'-rG:C and 3'-8-oxorG:C by LIG1 wild-type, respectively, and correspond to time points of 10, 30, 45, 60, 90, 120 sec. **(B)** Graph shows the time-dependent change in the amount of ligation products. Data points represent three independent replicates. Bar height is the mean, and error bars represent the SD. n.s., not significant; *P < 0.05 by ordinary two-way ANOVA with multiple comparisons.

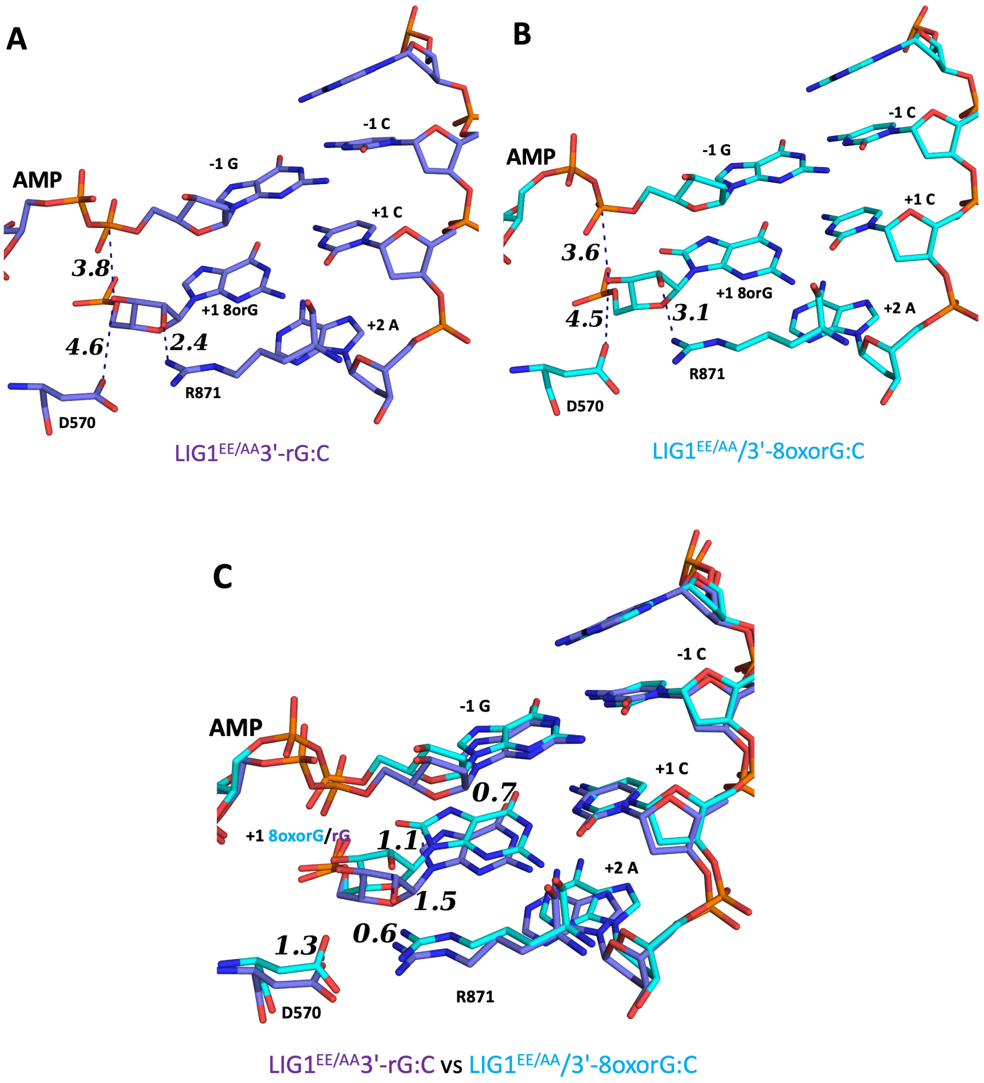

v

Supplementary Figure 10. Overlay of LIG1/RNA-DNA heteroduplexes with and without oxidatively damaged ribonucleotide. (A-C) Individual structures of LIG1$^{EE/AA}$/3'-rG:C and LIG1$^{EE/AA}$/3'-8-oxorG:C (A-B) and the overlay of both structures (C) show that 3'-8-oxorG:C adopts similar conformation with 3'-rG:C at the nick site. 3'-end of +1C nucleotide in the structure of 3'-8-oxorG:C moves closer to 5'-end resulting in a movement at 5'-PO_4 of -1G nucleotide relative to nick site. We previously solved LIG1$^{EE/AA}$/3'-rG:C structure (PDB:8VZL)

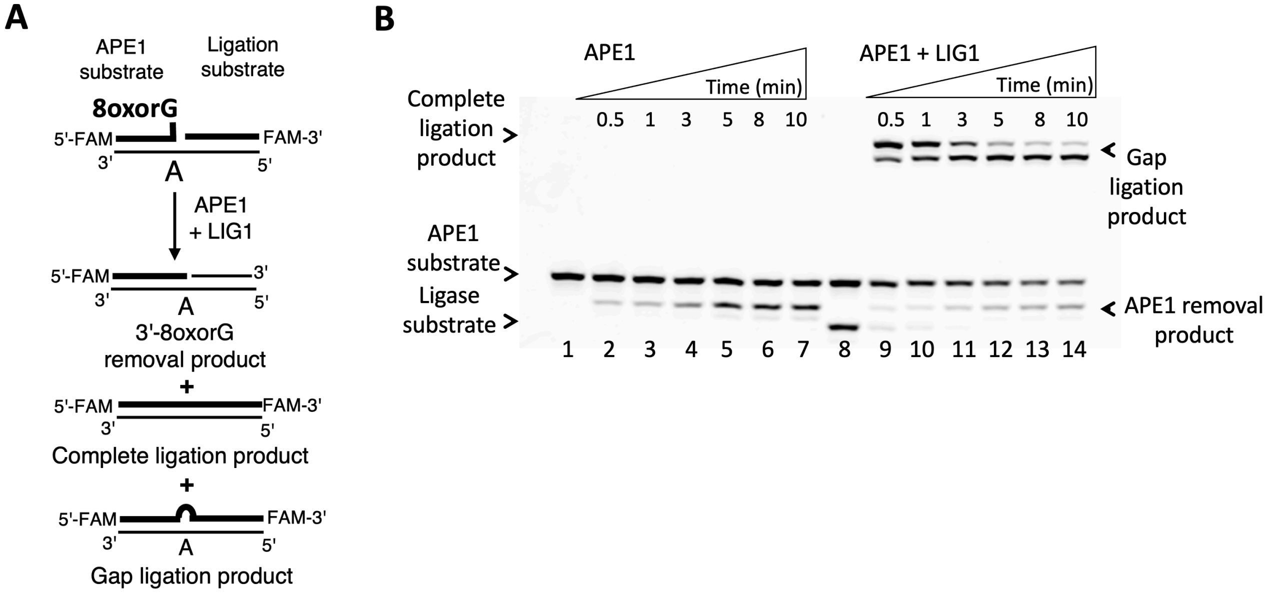

Supplementary Figure 11. Interplay between APE1 and LIG1 during processing of nick with 3'-8-oxorG:A. (A) Scheme shows reaction substrate and products observed in the repair assays in the presence of nick DNA substrate containing 3'-8-oxorG:A. **(B)** Lanes 1 and 8 are the negative enzyme controls of nick DNA substrates for APE1 and LIG1, respectively. Lanes 2-7 are 3'-8-oxorG removal products by APE1, and correspond to time points of 0.5, 1, 3, 5, 8, 10 min. Lanes 9-14 are the products of 3'-8-oxorG removal from template A by APE1 coupled to nick sealing by LIG1, and correspond to time points of 0.5, 1, 3, 5, 8, 10 min

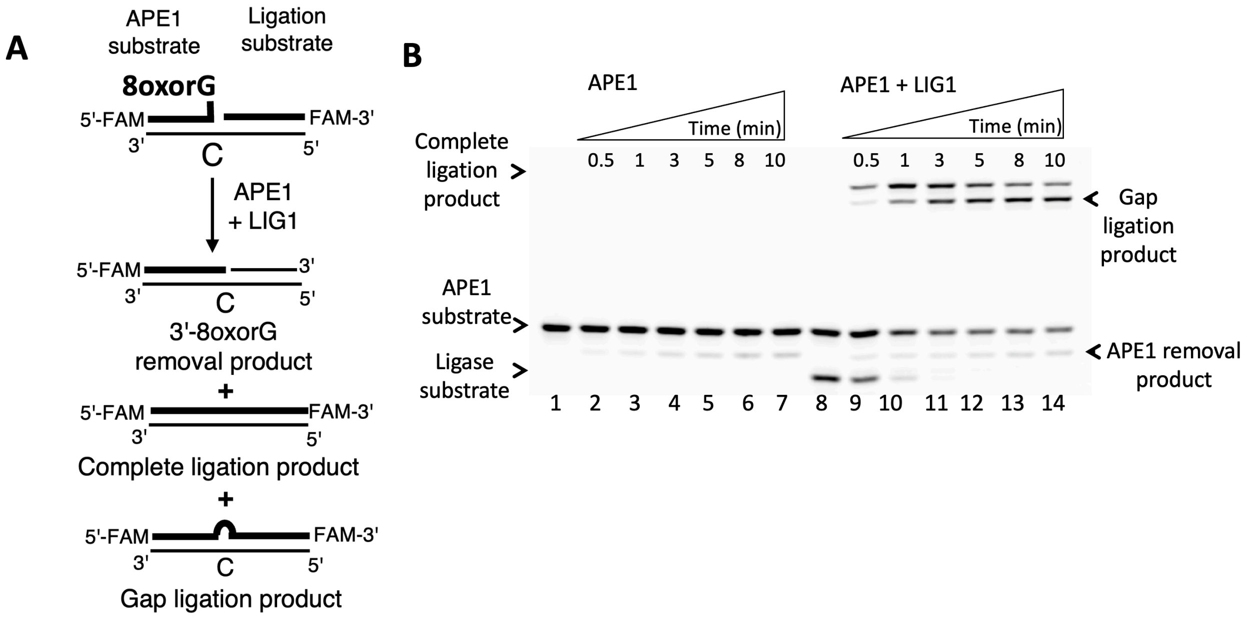

Supplementary Figure 12. Interplay between APE1 and LIG1 during processing of nick with 3'-8-oxorG:C. (A) Scheme shows reaction substrate and products observed in the repair assays in the presence of nick DNA substrate containing with 3'-8-oxorG:C. **(B)** Lanes 1 and 8 are the negative enzyme controls of nick DNA substrates for APE1 and LIG1, respectively. Lanes 2-7 are 3'-8-oxorG removal products by APE1, and correspond to time points of 0.5, 1, 3, 5, 8, 10 min. Lanes 9-14 are the products of 3'-8-oxorG removal from template C by APE1 coupled to nick sealing by LIG1, and correspond to time points of 0.5, 1, 3, 5, 8, 10 min.

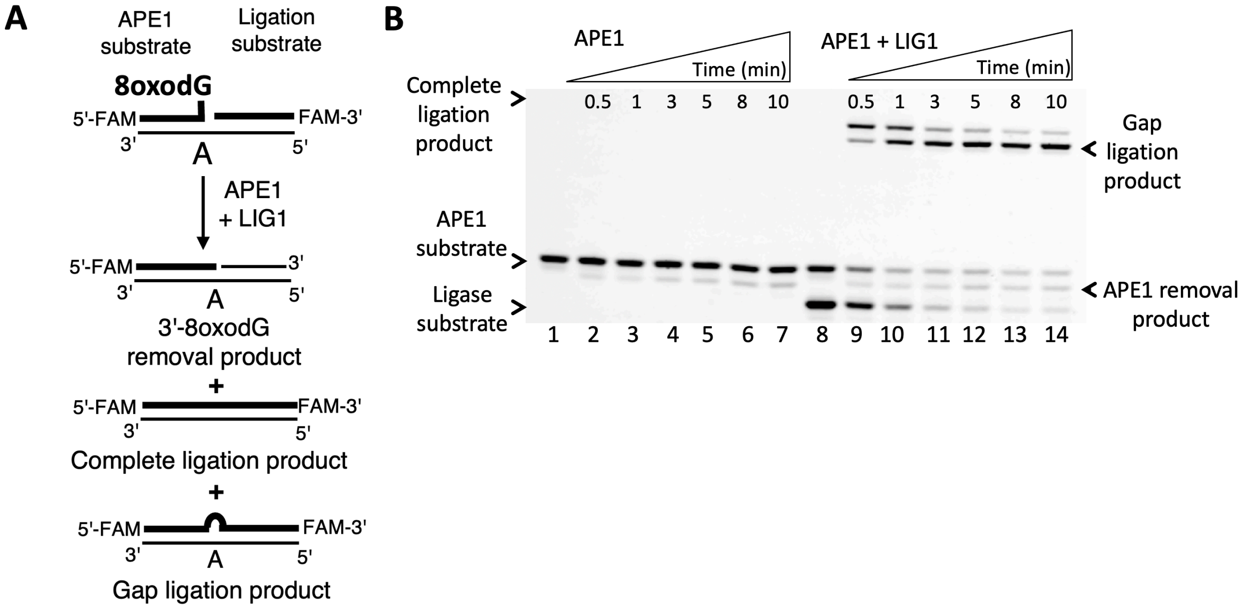

Supplementary Figure 13. Interplay between APE1 and LIG1 during processing of nick with 3'-8-oxodG:A. (A) Scheme shows reaction substrate and products observed in the repair assays in the presence of nick DNA substrate containing with 3'-8-oxodG:A. **(B)** Lanes 1 and 8 are the negative enzyme controls of nick DNA substrates for APE1 and LIG1, respectively. Lanes 2-7 are 3'-8-oxodG removal products by APE1, and correspond to time points of 0.5, 1, 3, 5, 8, 10 min. Lanes 9-14 are the products of 3'-8-oxodG removal from template A by APE1 coupled to nick sealing by LIG1, and correspond to time points of 0.5, 1, 3, 5, 8, 10 min.

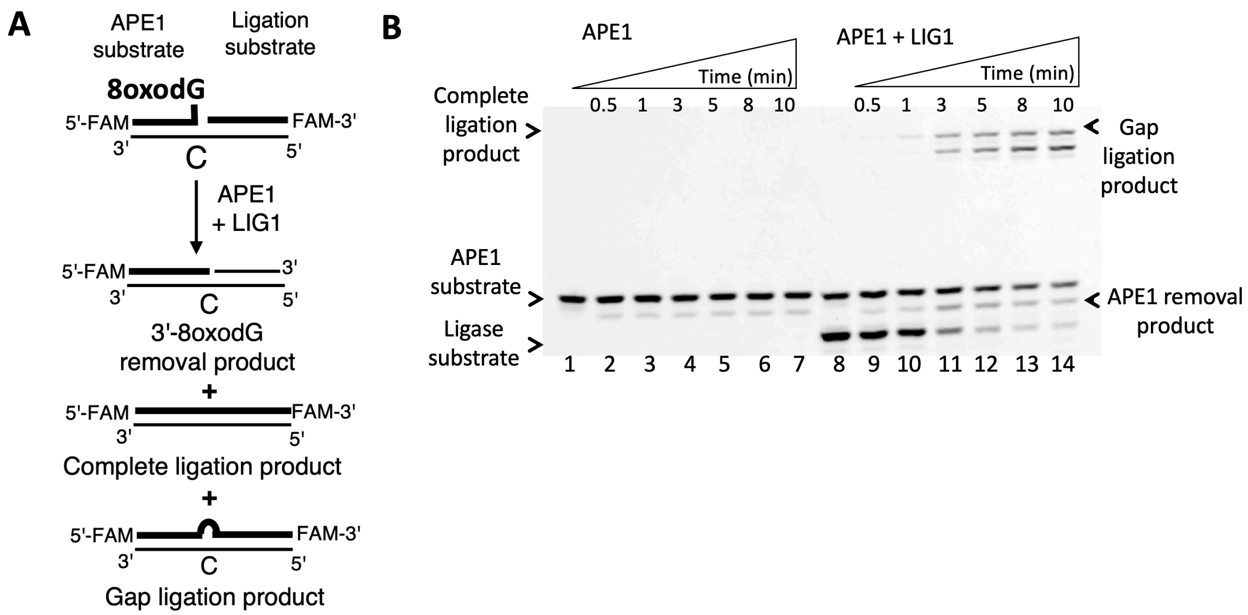

Supplementary Figure 14. Interplay between APE1 and LIG1 during processing of nick with 3'-8-oxodG:C. (A) Scheme shows reaction substrate and products observed in the repair assays in the presence of nick DNA substrate containing 3'-8-oxodG:C. **(B)** Lanes 1 and 8 are the negative enzyme controls of nick DNA substrates for APE1 and LIG1, respectively. Lanes 2-7 are 3'-8-oxodG removal products by APE1, and correspond to time points of 0.5, 1, 3, 5, 8, 10 min. Lanes 9-14 are the products of 3'-8-oxodG removal from template C by APE1 coupled to nick sealing by LIG1, and correspond to time points of 0.5, 1, 3, 5, 8, 10 min.

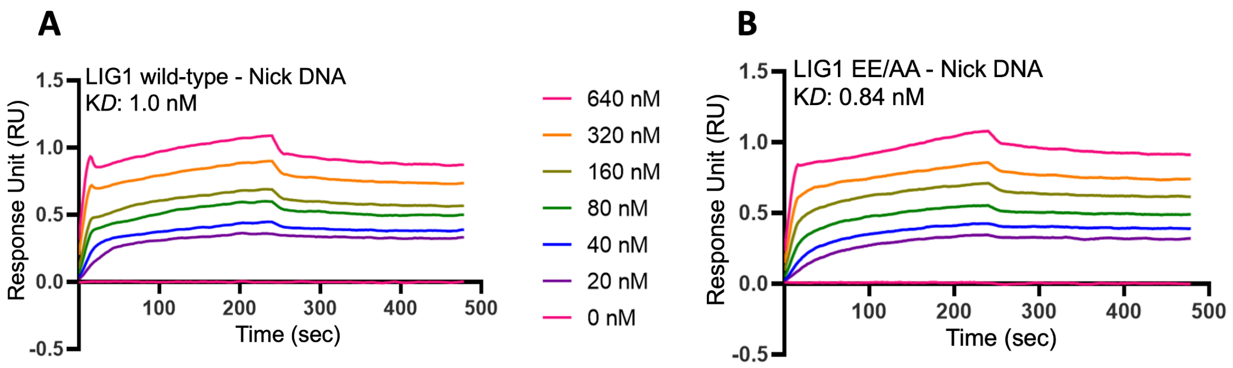

Supplementary Figure 15. Nick DNA binding modes of LIG1 wild-type and EE/AA mutant. **(A-B)** Nick DNA binding kinetics and the equilibrium binding constants (K_D) are shown for LIG1 wild-type (A) and low-fidelity mutant EE/AA (B). Sensorgrams are shown for the concentrations range of the proteins where the nick DNA with a biotin label is immobilized on the streptavidin biosensor.

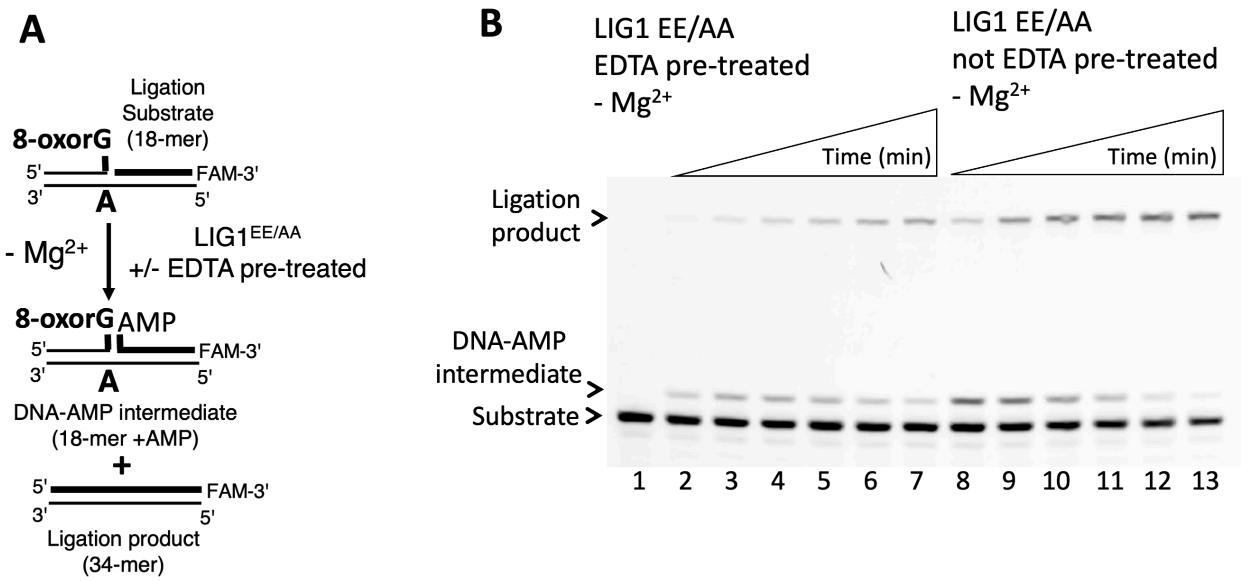

Supplementary Figure 16. Ligation of nick DNA substrate with 3'-8-oxorG:A by LIG1 low-fidelity mutant in the absence of Mg^{2+}. (A) Scheme shows reaction substrate and products observed in the ligation assays in the presence of the nick DNA substrate containing 3'-8-oxorG:A. **(B)** Line 1 is the negative enzyme control of the nick DNA substrate containing 3'-8-oxorG:A. Lanes 2-7 and 8-13 are the ligation products in the absence of Mg^{2+} by LIG1 EE/AA mutant purified with and without EDTA pre-treatment, respectively, and correspond to time points of 15, 30, 45, 60, 90, 120 min.

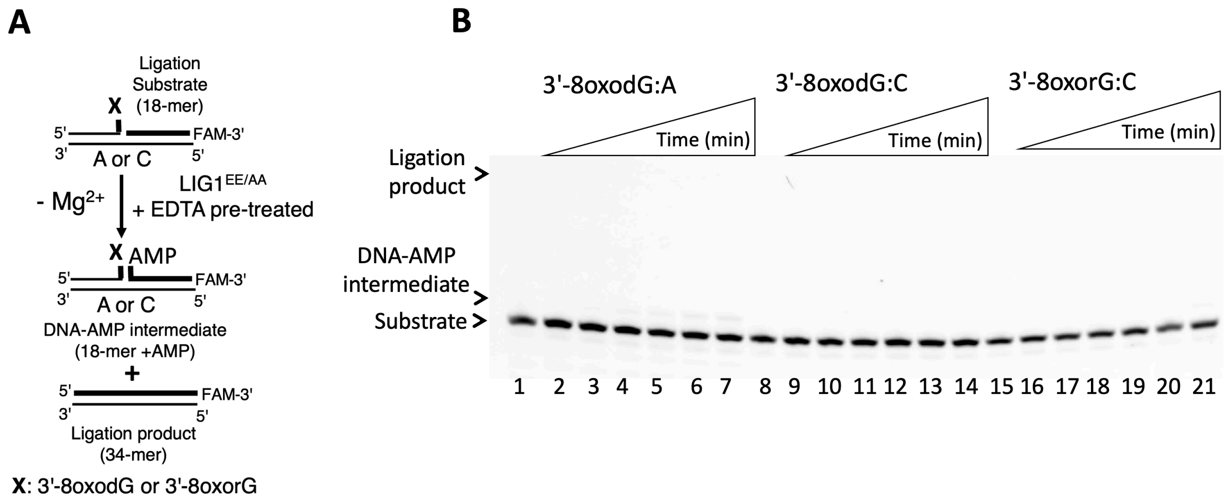

Supplementary Figure 17. Ligation of nick DNA substrates containing oxidatively damaged ends by LIG1 low-fidelity mutant in the absence of Mg^{2+}. (A) Scheme shows reaction substrate and products observed in the ligation assays. **(B)** Lanes 1, 8, and 15 are the negative enzyme controls of the nick DNA substrates containing 3'-8-oxodG:A, 3'-8-oxodG:C, and 3'-8-oxorG:C, respectively. Lanes 2-7, 9-14, and 16-21 are the ligation products in the presence of the nick DNA substrates containing 3'-8-oxodG:A, 3'-8-oxodG:C, and 3'-8-oxorG:C, respectively, in the absence of Mg^{2+} by LIG1 EE/AA mutant purified with EDTA pre-treatment, and correspond to time points of 15, 30, 45, 60, 90, 120 min.

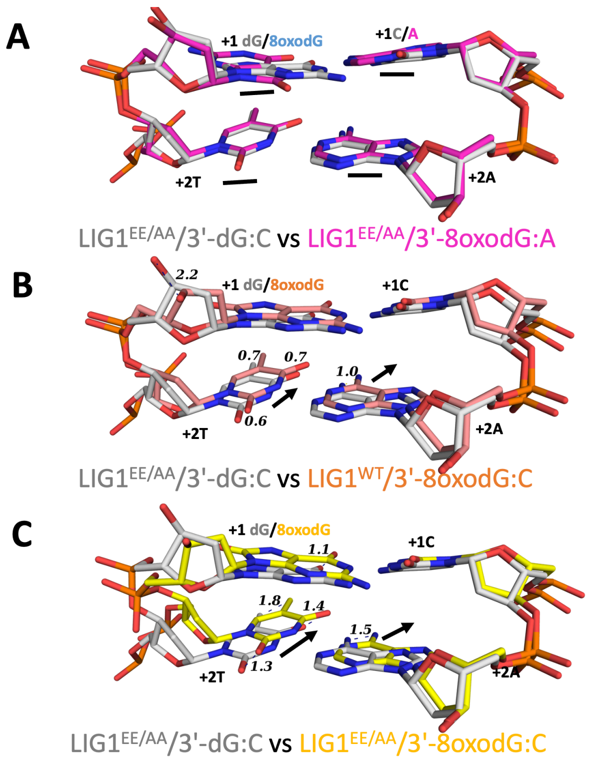

Supplementary Figure 18. Overlays of LIG1/nick complex structures with and without oxidatively damaged ends. (**A**) The overlay of LIG1$^{EE/AA}$/3'-dG:C and LIG1$^{EE/AA}$/3'-8-oxodG:A structures shows no misalignment in any of the nucleotides except 3'-8-oxodG and 3'-dG. (**B-C**) The overlays of LIG1^{WT}/3'-8-oxodG:C and LIG1$^{EE/AA}$/3'-8-oxodG:C structures with LIG1$^{EE/AA}$/3'-dG:C structure show that the misalignment in the deoxyribose of 3'-8-oxoG. +2 nucleotides show significant movement at +2 nucleotides T:A of LIG1^{WT}/3'-8-oxodG:C and LIG1$^{EE/AA}$/3'-8-oxodG:C structures, respectively. We previously reported structure of LIG1$^{EE/AA}$/3'-dG:C (PDB:8VDN).

Oligonucleotide	Sequence (5'-3')
Template A	GTCCGACAACGCATCAGC
Template C	GTCCGACCACGCATCAGC
Upstream X	GCTGATGCGT**X**
Downstream (5'-P)	P-GTCGGAC

Supplementary Table 1. Oligonucleotides used in LIG1 crystallization. Upstream oligonucleotides including a damaged base (X) at the 3'-end (8-oxodG or 8-oxorG), downstream oligo with a phosphate (P) at the 5'-end, and the oligonucleotide containing A or C at the template position were used to prepare the nick DNA substrates for LIG1 crystallization. The base at template base position is underlined and the base at the 3'-end of nick is shown in bold.

Protein	Nick	PDB	Crystal Conditions
LIG1$^{EE/AA}$	3'-8oxorG:A	9YHU	100 mM MES (pH 6.5), 200 mM Lithium acetate, 12% (w/v) PEG3350
LIG1$^{EE/AA}$	3'-8oxorG:C	9YHV	100 mM MES (pH 6.0), 150 mM Lithium acetate, 16% (w/v) PEG3350
LIG1$^{EE/AA}$	3'-8oxodG:A	9YHW	100 mM MES (pH 6.5), 100 mM Lithium acetate, 12% (w/v) PEG3350
LIG1$^{EE/AA}$	3'-8oxodG:C	9YHX	100 mM MES(pH 6.2), 200 mM Lithium acetate, 16% (w/v) PEG3350
LIG1^{WT}	3'-8oxodG:C	9YHY	100 mM MES(pH 6.2), 200 mM Lithium acetate, 16% (w/v) PEG3350

Supplementary Table 2. Crystalization conditions of LIG1/nick DNA complexes.

Nick DNA Substrates	Sequences
3'-dG:C	5’-CATGGGCGGCATGAACC**G**GAGGCCCATCCTCACC-3’-FAM<br>3’-GTACCCGCCGTACTTGGCCTCCGGGTAGGAGTGG-5’
3'-8oxodG:A	5’-CATGGGCGGCATGAACC**X**GAGGCCCATCCTCACC-3’-FAM<br>3’-GTACCCGCCGTACTTGGACTCCGGGTAGGAGTGG-5’
3'-8oxodG:C	5’-CATGGGCGGCATGAACC**X**GAGGCCCATCCTCACC-3’-FAM<br>3’-GTACCCGCCGTACTTGGCCTCCGGGTAGGAGTGG-5’
3'-8oxorG:A	r<br>5’-CATGGGCGGCATGAACC**X**GAGGCCCATCCTCACC-3’-FAM<br>3’-GTACCCGCCGTACTTGGACTCCGGGTAGGAGTGG-5’
3'-8oxorG:C	r<br>5’-CATGGGCGGCATGAACC**X**GAGGCCCATCCTCACC-3’-FAM<br>3’-GTACCCGCCGTACTTGGCCTCCGGGTAGGAGTGG-5’

Supplementary Table 3. Nick DNA substrates used in ligation assays. FAM denotes a fluorescence tag at 3'-end of nick DNA substrates. The base at the 3'-end is shown as bold and the template base is underlined. X is 8-oxodG or 8-oxorG.

Nick DNA Substrates	Sequences
3'-8oxodG:A	FAM-5’-CATGGGCGGCATGAACC**X**GAGGCCCATCCTCACC-3’<br>3’-GTACCCGCCGTACTTGGACTCCGGGTAGGAGTGG-5’
3'-8oxodG:C	FAM-5’-CATGGGCGGCATGAACC**X**GAGGCCCATCCTCACC-3’<br>3’-GTACCCGCCGTACTTGGCCTCCGGGTAGGAGTGG-5’
3'-8oxorG:A	r<br>FAM-5’-CATGGGCGGCATGAACC**X**GAGGCCCATCCTCACC-3’<br>3’-GTACCCGCCGTACTTGGACTCCGGGTAGGAGTGG-5’
3'-8oxorG:C	r<br>FAM-5’-CATGGGCGGCATGAACC**X**GAGGCCCATCCTCACC-3’<br>3’-GTACCCGCCGTACTTGGCCTCCGGGTAGGAGTGG-5’

Supplementary Table 4. Nick DNA substrates used in APE1 exonuclease assays. FAM denotes a fluorescence tag at 5'-end of the nick DNA substrates. The base at the 3'-end is shown as bold and the template base is underlined. X is 8-oxodG or 8-oxorG.

Nick DNA Substrates	Sequences
3'-8oxodG:A	FAM-5'-CATGGGCGGCATGAACC**X**GAGGCCCATCCTCACC-3'-FAM<br>3'-GTACCCGCCGTACTTGGACTCCGGGTAGGAGTGG-5'
3'-8oxodG:C	FAM-5'-CATGGGCGGCATGAACC**X**GAGGCCCATCCTCACC-3'-FAM<br>3'-GTACCCGCCGTACTTGGCCTCCGGGTAGGAGTGG-5'
3'-8oxorG:A	r<br>FAM-5'-CATGGGCGGCATGAACC**X**GAGGCCCATCCTCACC-3'-FAM<br>3'-GTACCCGCCGTACTTGGACTCCGGGTAGGAGTGG-5'
3'-8oxorG:C	r<br>FAM-5'-CATGGGCGGCATGAACC**X**GAGGCCCATCCTCACC-3'-FAM<br>3'-GTACCCGCCGTACTTGGCCTCCGGGTAGGAGTGG-5'

Supplementary Table 5. Nick DNA substrates used in APE1 and LIG1 assays. FAM denotes a fluorescence tag at both 3'- and 5'-ends of the nick DNA substrates. The base at the 3'-end is shown as bold and the template base is underlined. X is 8-oxodG or 8-oxorG.

DNA substrate	Sequence
Nick	5'-CATGGGCGGCATGAACCGGAGGCCCATCCTCACC-3'-Biotin<br>3'-GTACCCGCCGTACTTGGCCTCCGGGTAGGAGTGG-5'

Supplementary Table 6. Nick substrate used in DNA binding measurements. Nick DNA substrate containing 3'-dG:C and 3'-Biotin was used in BLI-based DNA binding measurements of LIG1 wild-type and EE/AA mutant. The base at the template position is underlined.

RMSD (Å)	LIG1$^{EE/AA}$/ 3'-8oxorG:A Step 3 9YHU	LIG1$^{EE/AA}$/ 3'-8oxorG:C Step 2 9YHV	LIG1$^{EE/AA}$/ 3'-8oxodG:A Step 2 9YHW	LIG1$^{EE/AA}$/ 3'-8oxodG:C Step 2 9YHX	LIG1^{WT}/ 3'-8oxodG:C Step 2 9YHY	LIG1$^{EE/AA}$/ 3'-rG:C Step 2 8 VZL	LIG1$^{EE/AA}$/ 3'-rG:C Step 3 8VDS	LIG1$^{EE/AA}$/ 3'-dG:C Step 2 8VDN	LIG1$^{EE/AA}$/ 3'-8oxodG:A Step 2 6P0E
LIG1$^{EE/AA}$/3'-8oxorG:A Step 3, 9YHU		0.601(556)	0.265(553)	0.524(585)	0.598(571)	0.475(589)	0.608(602)	0.550(603)	0.471(594)
LIG1$^{EE/AA}$/3'-8oxorG:C Step 2, 9YHV			0.585(542)	0.297(533)	0.321(542)	0.631(565)	0.462(545)	0.491(546)	0.734(566)
LIG1$^{EE/AA}$/3'-8oxodG:A Step 2, 9YHW				0.488(565)	0.557(558)	0.538(586)	0.697(597)	0.580(588)	0.363(564)
LIG1$^{EE/AA}$/3'-8oxodG:C Step 2, 9YHX					0.280(570)	0.568(587)	0.403(551)	0.420(566)	0.669(606)
LIG1^{WT}/3'-8oxodG:C Step 2, 9YHY						0.630(572)	0.475(551)	0.475(543)	0.716(584)
LIG1$^{EE/AA}$/3'-rG:C Step 2, 8VZL							0.391(592)	0.334(595)	0.522(588)
LIG1$^{EE/AA}$/3'-rG:C Step 3, 8VDS								0.242(584)	0.691(614)
LIG1$^{EE/AA}$/3'-dG:C Step 2, 8VDN									0.614(594)

Supplementary Table 7. The root mean square deviation (RMSD) of LIG1 structures solved previously and presented in this study. The values in the parenthesis represents no of atoms aligned against.

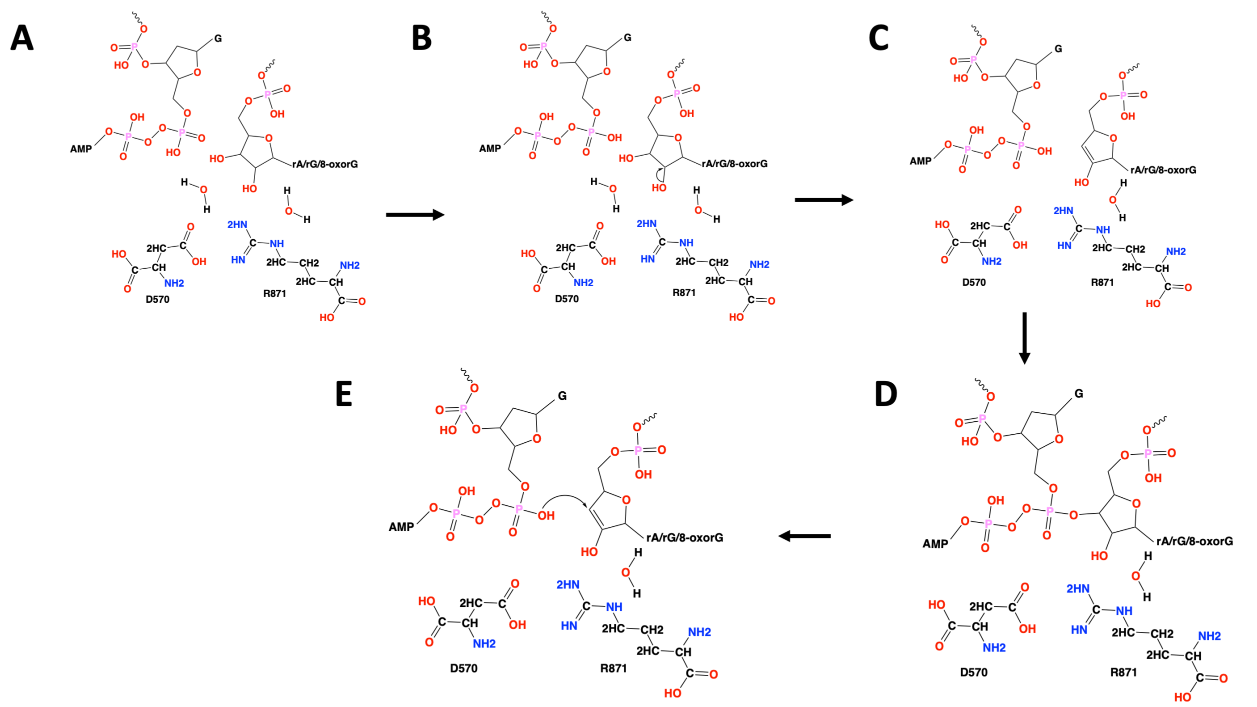

Supplementary Scheme 1. Potential reaction leading to a lack of oxidatively damaged sugar discrimination at the 3'-end of nick by LIG1. (A-E). The phosphate diester bond can form a condensation reaction between 3'-OH and 5'-PO_4 ends of the nick. In the post-catalytic step structures, the water bridge between D570 and the 3'-OH, along with the interaction between the 2'-OH and the R871 side chain, helps with establishing a chemical conformation that is favorable for the reaction (A). The interaction with R871 side chain causes electron localization at the 2'-C (B), promoting the formation of a π-bond between the 2' and 3' carbons of -1rA/G at the nick (C). Meanwhile, a water molecule positioned between the 3'-OH of +1rA/rG/8-oxorG and side chain D570 facilitates the departure of a water leaving group from the 3'-carbon of +1 rA/rG/8-oxorG, generating a carbocation at the 3'-end (D). This sets the stage for a nucleophilic attack by the OH group of the 5'-PO_4 of +1G at the nick, leading to the formation of a phosphate linkage between the 5'-PO_4 of +1G and the 3'-C of +1 rA/rG/8-oxorG through an oxygen bridge (E).
